# Promyelocytic Leukemia Restricts Enterovirus 71 Replication by Inhibiting Autophagy

**DOI:** 10.3389/fimmu.2018.01268

**Published:** 2018-06-05

**Authors:** Deyan Chen, Chunhong Feng, Xiaoyan Tian, Nan Zheng, Zhiwei Wu

**Affiliations:** ^1^Center for Public Health Research, Medical School, Nanjing University, Nanjing, China; ^2^State Key Laboratory of Analytical Chemistry for Life Science, Nanjing University, Nanjing, China; ^3^Medical School, Jiangsu Key Laboratory of Molecular Medicine, Nanjing University, Nanjing, China

**Keywords:** promyelocytic leukemia, promyelocytic leukemia protein-nuclear bodies, enterovirus 71, autophagy, 3Cpro, interferon-β

## Abstract

The promyelocytic leukemia (PML) protein, also known as TRIM19, functions as a major organizer of PML nuclear bodies (NBs) in most mammalian cells and plays important roles in antiviral activities against both DNA and RNA viruses. In this study, we found that the downregulation of PML rendered HeLa cells more susceptible to infection by enterovirus 71 (EV71), and the overexpression of the PMLIII or PMLIV isoforms inhibited viral protein expression and resulted in viral titers that were 2–3 log units lower than those in the control. Using short interfering RNAs, the downregulation of either the PMLIII or PMLIV isoform increased both viral protein VP1 expression and viral production. The PML repression of EV71 replication was partially mediated by the inhibition of autophagy, and PML deficiency triggered autophagy. Furthermore, the EV71 infection resulted in a reduction in PML independent of the proteasome pathway. Instead, PML degradation was mediated by virus protease 3C^pro^. In conclusion, PML contributes to a cellular antiviral effect by inhibiting autophagy, which is countered by a disruption of promyelocytic leukemia protein-nuclear bodies mediated by viral protease 3C^pro^.

## Introduction

Promyelocytic leukemia protein-nuclear bodies (PML-NBs) are dynamic cellular structures consisting of numerous transiently and permanently localized proteins. Promyelocytic leukemia (PML), also known as TRIM19, MYL, PP8675, or RNF71, is the major component of PML-NBs and plays important roles in genome stability, programmed cell death, and antiviral activities (1). PML consists of seven isoforms, PMLI–VII, derived from alternative splicing of a single gene (2). All isoforms share a similar N-terminal region encoded by exons 1–3 and containing the RBCC motif through which PML multimerizes to form a ring-like structure that binds to the nuclear matrix, forming PML-NBs (1). The C-terminal region varies and determines its different biological functions (3). The PML promoter region contains targets for STATs, IRFs, and p53, and the PML gene is directly inducible by interferons (IFNs) including type I and type II, leading to an increased expression of PML isoforms, increased numbers, and bigger size of PML-NBs (2). The other major PML-NB components include Sp100, Daxx, and ATRX and they act as intrinsic restriction factors that repress various viral replications (4–7). PML can be SUMOylated, which enables its interaction with other SUMOylated proteins and itself. PML SUMOylation was needed to recruit other PML-NB components and to maintain the biogenesis of PML nuclear bodies (NBs) (3, 8).

Promyelocytic leukemia has been extensively characterized as the first cellular defense against herpes infections (4, 5). The antiviral effects of PML were initially suggested based on the following evidence: (I) IFN treatment including type I and type II could lead to the increased expression of PML and the high numbers and the bigger size of PML NBs in the IFN-treated cells (2, 6); (II) viral infection often resulted in a disruption or distortion of the PML-NB structure. For example, PML-NB constituents may be degraded following HSV-1 and human cytomegalovirus (HCMV) infections, which resulted in the disruption of PML-NBs (7, 9), whereas PML-NB deformation and reorganization were observed in adenovirus and papillomavirus infections, respectively (10, 11); and (III) PML KO mice were prone to infections (12). However, viruses have evolved ways to evade the antiviral activities of PML (4–7). The ICP0 protein of HSV-1 can act as an E3 ubiquitin ligase and cause the degradation of SUMOylated PML (13). The immediate early protein IE1 of HCMV can specifically affect the SUMOylation of PML independent of the proteasome pathway (14).

Evidence also showed that in addition to DNA viruses, PML exhibited inhibitory activity against RNA viruses. PML KO mice become sensitive to infection including vesicular stomatitis virus (VSV), rabies virus, and arenavirus lymphocytic choriomeningitis virus (LCMV) (15–17). In a recent report, the antiviral activity of PML was observed in dengue virus (DENV)-2 infected-A549 cells (18). However, PML activity in enterovirus 71 (EV71) infection has not been documented to date. In this study, we demonstrated that downregulation of total PML increases both viral protein VP1 expression and viral production and that the overexpression of the PMLIII and PMLIV isoforms rendered cells resistant to EV71 infection. PML mediated antiviral activity against EV71 infection by inhibiting autophagy in the infected cells. EV71 infection induced PML degradation, which was mediated by viral 3C protease independent of the proteasome pathway.

## Materials and Methods

### Reagents, Cell Lines, Plasmids, and Viruses

The viral protease 3C^pro^ inhibitor rupintrivir was purchased from Sigma (St. Louis, MO, USA). The proteasome inhibitors epoxomicin (Selleckchem, Houston, TX, USA) and MG132 (Selleckchem, Houston, TX, USA) were treated with cells 1 h following EV71 infection, and the inhibitors were always maintained in the process. Epoxomicin and MG132 were used at 1 µM and 100 nM, respectively. Goat anti-mouse IgG (H + L) with Alexa Fluor 488, SYBR, DAPI, and antibody specific for GFP were from Life Technologies (Carlsbad, CA, USA). The antibody specific for ATG5 was obtained from Proteintech Group (IL, USA). IRDye 800 goat-anti-mouse and IRDye 680 goat-anti-rabbit were purchased from LI-COR (Lincoln, NE, USA). Mouse anti-GAPDH antibody and lysis buffer RIPA were obtained from Santa Cruz (Santa Cruz, CA, USA). Mouse anti-FLAG and rabbit anti-LC3B (Cat. No. L7543) antibodies were obtained from Sigma (St. Louis, MO, USA). Antibodies specific for PML (ab72137), PML (ab96051), and VP1 (ab36367) were obtained from Abcam (Cambridge, UK). Anti-VP1 antibody (GTX132338) was purchased from GeneTex. Recombinant human IFN-β was obtained from Sino Biological Inc. (Cat. No. 10704-H02H-20). PML small interfering RNA (siRNA) (sc-36284) was purchased from Santa Cruz (Santa Cruz, CA, USA). Atg5 siRNA (#6345) and a non-specific scrambled siRNA (#6586) were obtained from CST (Cell Signaling Technology, USA).

Rhabdomyosarcoma (RD) cells, originally obtained from American Type Culture Collection (ATCC) (Manassas, VA, USA), were purchased from Cell Bank of Chinese Academy of Sciences (CAS) (Shanghai, China). HeLa, mouse embryonic fibroblast (MEF), HEK293T, and Vero cells were obtained from ATCC (Manassas, VA, USA). The PML^−/−^ human cervical cancer (HeLa) cell line (19) and PML^−/−^ MEFs cell line (20) were gifts from Professor Jun Tang (China Agricultural University, Beijing). Both PML^−/−^ HeLa and MEF cells were generated from corresponding WT cells obtained from ATCC. The cells were cultured in DMEM high glucose containing 10% FBS (Life Technologies, Carlsbad, CA, USA). EV71 BrCr strain was a kind gift from Professor Bin Wu, Jiangsu Provincial Centers of Disease Control; EV71 Fuyang0805 strain was from Professor Erguang Li, Nanjing University. They were propagated on RD cells. Confluent RD cells maintained in DMEM containing 2% FBS were inoculated with the viruses at a multiplicity of infection (MOI) of 0.2. The viral stocks were collected from the supernatants of infected RD cells 2 days post-infection (p.i.) and titrated on Vero cells by plaque assay and EV71 infection was carried out as described previously (21). Gluc-EV71 that carried a Gaussia luciferase reporter gene in EV71 genome was obtained from Dr. Bo Zhang, Wuhan Institute of Virology, CAS (Wuhan, China). The Gaussia luciferase activity was determined by using BioLux Gaussia luciferase assay kit (New England Biolabs, Beverly, MA, USA).

Plasmid pEGFP-3C was a gift from Dr. Xiaobo Lei and Professor Jianjun Wang, as described elsewhere (22). Plasmid expressing pEGFP-PMLI–VI and flag-PMLI–VI were kind gifts from Professor Jun Tang (China Agricultural University, Beijing), as described elsewhere (20). The plasmid pEGFP-N3-2A was constructed by cloning 2A^pro^ coding sequence of EV71 Fuyang0805 strain into pEGFP-N3 vector (Clontech, Palo, CA, USA).

### RNA Interference and DNA Transfection

Cells were transfected with 2.5 µg of plasmid DNA or siRNA in 6-well plate by using Lipofectamine 3000 and Lipofectamine RNAi Max transfection reagents (Invitrogen), respectively, according to the manufacturer’s recommendations. The siRNA specific for PML (to all PML isoforms) was purchased from Santa Cruz (sc-36284). The siRNA sequences were reported previously (23). The siRNA scramble sequence was 5′-GCAUGAACCGAGGCCCAUUU-3′ and served as a control. The siRNA specific for the PMLIII isoform: 5′-AGUGCAUGGAGCCCAUGGATT-3′. The siRNA specific for the PMLIV isoform: 5′-UGAAAGUGGGUUCUCCUGGTT-3′.

HeLa or 293T cells were transiently transfected with plasmids pEGFP-PML (I–VI) or the empty plasmid pEGFP-C1 using Lipofectamine 3000 (Life Technologies) for 24 h followed with mock infection or infection with EV71 (MOI = 5). After 24 h, cultural medium was removed, and the cells were washed with ice cold PBS. Total protein was prepared by lysing the cells with RIPA buffer and analyzed by Western blot. The autophagic process was triggered by culturing cells in medium without serum for 2 h (Earle’s balanced salt solution). Autophagy was inhibited by treating the cells with 3-methyladenine (3-MA) (250 µM) in DMEM with 10% FBS (24, 25).

The PML^+/+^ or PML^−/−^ HeLa cells in 80% confluence were transfected with a plasmid expressing GFP-LC3 using Lipofectamine 3000. 12 h later, the cells were infected with EV71 (MOI = 5), and the GFP-LC3 punctate aggregations were counted under a fluorescence microscope. Cells containing two or more GFP-LC3 punctate aggregations were defined as autophagy positive. The number of autophagy-positive cells relative to GFP-expressing cells was considered as the significant differences. The samples were examined under a fluorescence microscopy, and images were acquired by using a confocal microscope Olympus FluoView FV10i (Tokyo, Japan). Data points presented in the text are from four different fields.

### Western Blot

Proteins were extracted from cells in ice cold RIPA lysis buffer (Santa Cruz), and total protein concentrations were determined using BCA protein assay kit (Pierce, Rockford, IL, USA). Total proteins were separated on SDS-PAGE and transferred to PVDF membranes. Proteins were detected by antibodies against PML (ab36367, 1:1,000; ab96051, 1:500), mouse-anti-FLAG antibody (1:1,000), mouse-anti-VP1 antibody (1:1,000), rabbit-anti-p62 (1:1,000), rabbit-anti-LC3B (1:1,000), mouse-anti-GFP (1:1,000) or rabbit-anti-GAPDH (1:2,000), and IRDye IgG (1:10,000) for 30 min. Bands were visualized under Li-COR Odyssey Infrared Imager (Li-COR).

### Immunofluorescence Analysis

HeLa cells were grown in F10mm coverslips in 24-well plates overnight. Plasmids expressing pEGFP-PMLIII, pEGFP-PMLIV, and the empty plasmid pEGFP-C1 were transfected into the cells with Lipofectamine 3000. 24 h post transfection, the cells were infected with EV71 (MOI = 5) for 24 h. The cells were then washed three times with PBS before being fixed with 4% paraformaldehyde for 15 min at room temperature and then permeabilized with 0.1% Triton X-100 for 10 min. The slides were washed three times with 0.1% PBS-Tween, blocked with blocking buffer (4% dry milk without fat), stained with a mouse anti-VP1 antibody (ab36367, Abcam), and followed with a goat-anti-mouse-Alexa 594 (1:1,000 dilution). Nuclei were stained with DAPI at 1 μg/ml (Sigma-Aldrich). Fluorescent images were acquired using an Olympus FluoView FV10i (Tokyo, Japan) confocal microscope.

### RNA Extraction and PCR

Quantification of PML, PMLIII, and PMLIV was performed using reverse transcription-PCR with the following oligonucleotide pairs as primers: 5′-CATCACCCAGGGGAAAGATG-3′ and 5′-GGTCAACGTCAATAGGGTCC-3′ for PML; 5′-CCCGTCATACGAAGTGAGGT-3′ and 5′-AGACTGAGGGCTGGAAGAGA-3′ for PMLIII; 5′-TGGACGAGAACCTTGCTGAC-3′ and 5′-CCAGGAGAACCCACTTTCAT-3′ for PMLIV; 5′-AGCTCACTGGCATGGCCTTC-3′ and 5′-ACGCCTGCTTCACCACCTTC-3′ for GAPDH. The PCR conditions were carried out as described previously (26).

### Statistics

Data from three independent experiments were showed as mean ± SEM by using GraphPad Prism 5. Data comparison was achieved with a two-tailed Student’s *t*-tests or one-way ANOVA followed by Student–Newman–Keuls tests. The significance was determined by Student’s *t*-tests. ***p < 0.005, **p < 0.01, and *p < 0.05 were considered significant.

## Results

### EV71 Replication Increased in the Absence of PML

**Figure 1:**
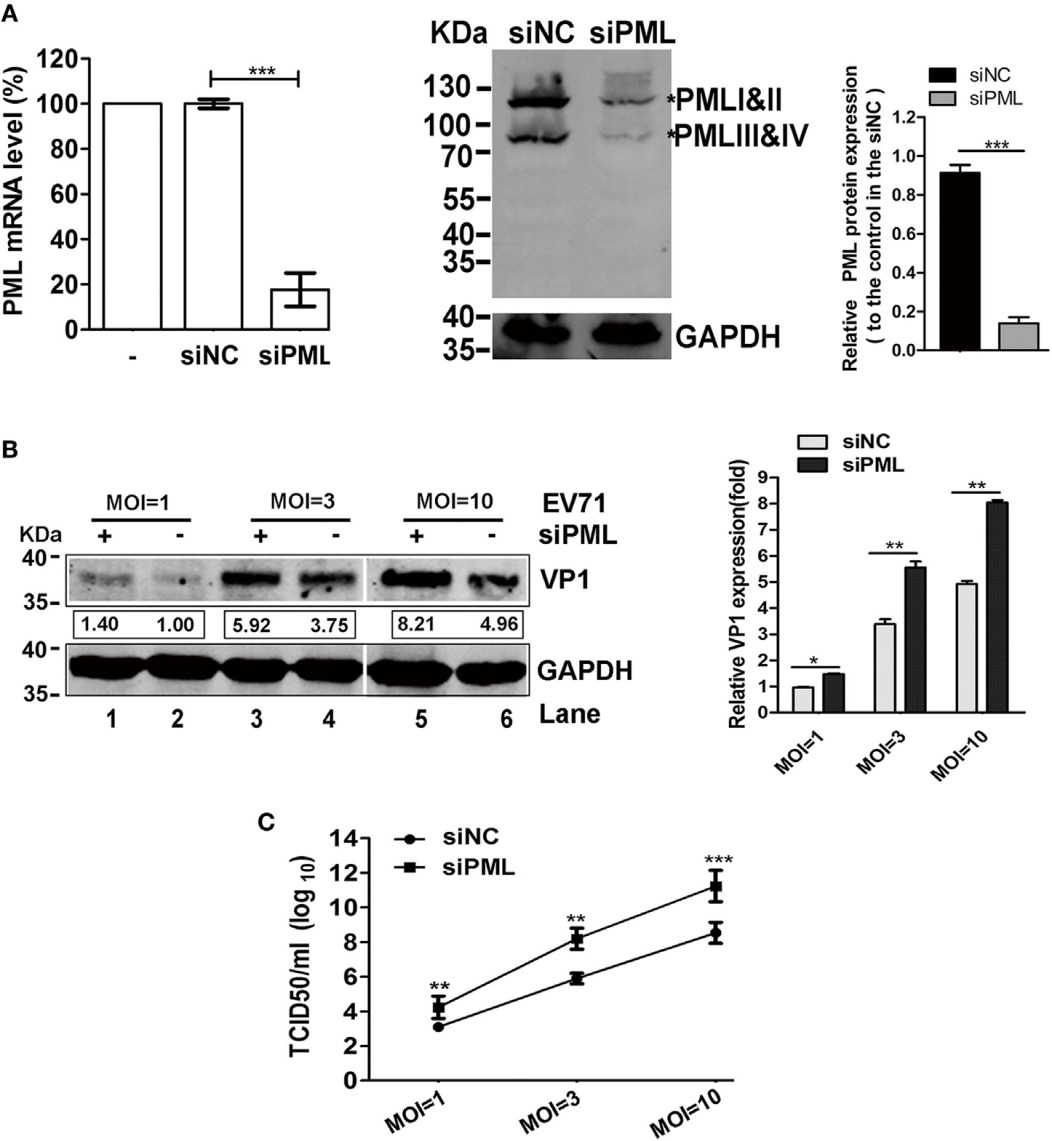
Promyelocytic leukemia (PML) depletion increased enterovirus 71 (EV71) replication in HeLa cells. **(A)** Efficacy of PML downregulation by siPML in HeLa cells. HeLa cells were treated with either a scrambled sequence (siNC) or siPML (common to all PML isoforms), and the PML mRNA transcripts were measured by performing real-time PCR using primers specific for PML. The data are presented as the mean ± SEM of three independent experiments (***p < 0.001). Cell lysates from the same siNC- or siPML-treated HeLa cells were analyzed by performing a Western blot analysis using antibodies against PML and GAPDH (loading control). Bands with a red asterisk indicate the different PML isoforms (PMLI and II have apparent molecular weights of 117 kDa and co-migrate as a single band, while PMLIII and IV have apparent molecular weights of 78 kDa and co-migrate). The total amount of PML proteins was quantified by performing a densitometry scan of all isoforms and was first normalized to GAPDH and then to the siNC control. A representative result based on three independent experiments is shown. The value of the mock treatment is set as 1.00 (100%) (***p < 0.001). **(B)** VP1 increased in the PML-silenced HeLa cells. HeLa cells in 6-well plates were transfected with siNC or siPML and infected with EV71 FY0805 at various multiplicity of infections (MOIs) 24 h after transfection. VP1 was determined by performing a Western blot analysis of the cell lysates. Densitometry was performed, and the values from three independent experiments were averaged. The data are expressed as the fold change in VP1 expression normalized to siNC at an MOI of 1 from three independent experiments. siNC treatment at an MOI of 1 is assigned the value of 1.00. **(C)** Viral titers in the supernatants were titrated using Vero cells at 24 h post-infection. The data are expressed as the mean ± SEM of three separate experiments (*p < 0.05, **p < 0.01, ***p < 0.001).

Promyelocytic leukemia was reported to have antiviral activities against a number of RNA viruses, such as the LCMV, rabies virus, poliovirus, encephalomyocarditis virus (EMCV), VSV, and DENV (13, 14, 18, 27, 28). To investigate the roles of PML in the replication of EV71, we determined the effects of the down-modulation of endogenous PML on EV71 replication. Using a pool of siRNAs targeting all six isoforms of PML, we efficiently downregulated PML expression in HeLa cells by 80% compared with that in the siNC-treated cells, as confirmed by both qPCR and Western blot analyses (Figure 1A). The effect of PML on EV71 replication and viral production was assessed by performing a TCID_50_ assay and Western blot analysis for viral protein VP1, respectively. As shown in Figure 1B, VP1 expression in the PML-knockdown HeLa cells was significantly higher than that in the siNC-treated cells for various MOIs (Figure 1B, Lanes 1, 3, and 5 compared with Lanes 2, 4, and 6, respectively). To determine the effect of the downregulation of PML on viral production, the cultural supernatants were collected for TCID_50_ determination in Vero cells. As shown in Figure 1C, at MOIs of 3 and 10, the viral production in the PML-knockdown HeLa cells was 100- and 1,000-fold higher than that in the siNC-treated cells, respectively. Altogether, PML conferred cellular resistance to EV71 infection.

**Figure 2:**
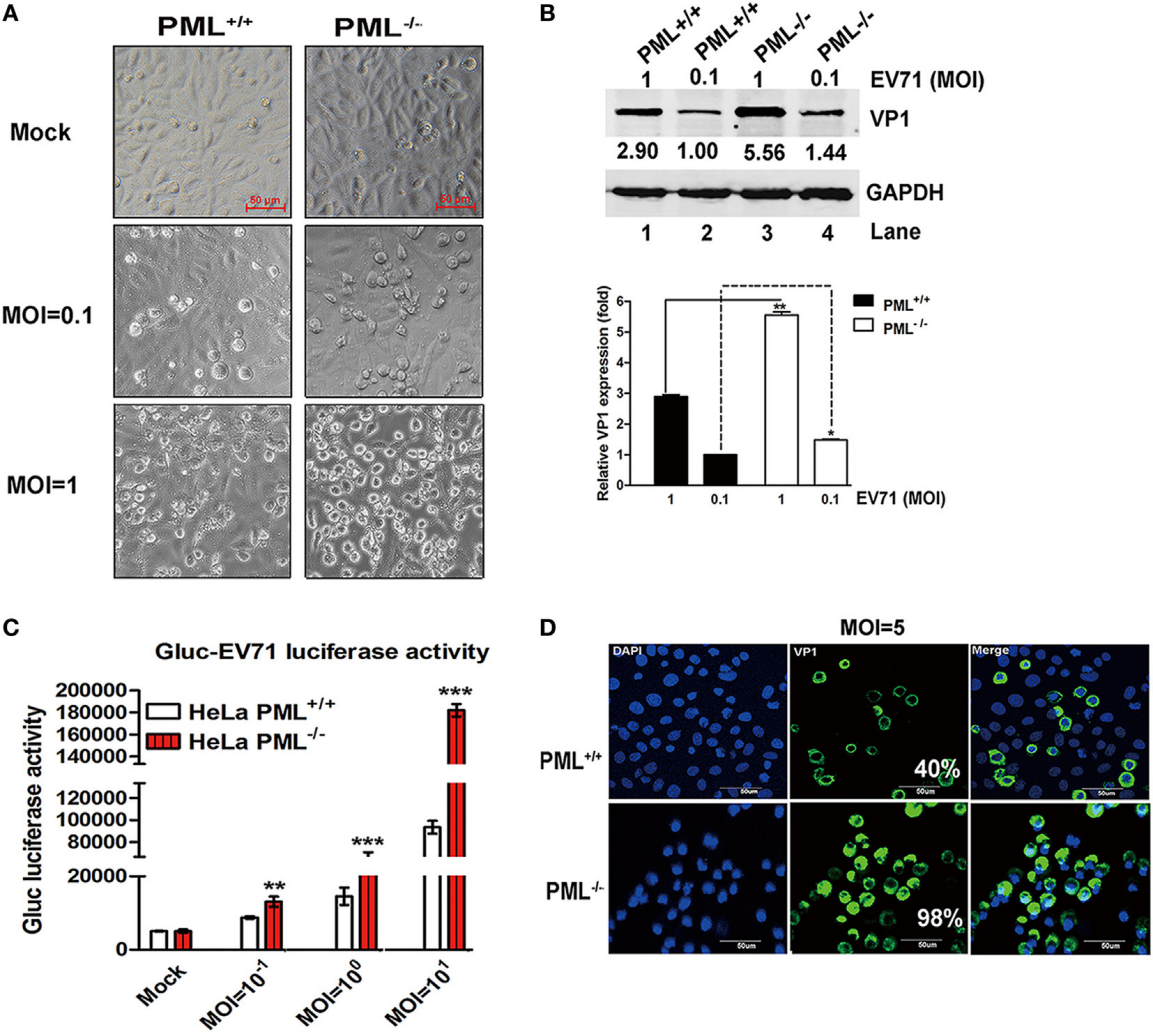
Comparison of enterovirus 71 (EV71) infection in PML^−/−^ and PML^+/+^ cells. **(A)** PML^+/+^ and PML^−/−^ cells grown in 12-well plates were infected with Gluc-EV71 at various multiplicity of infections (MOIs) (mock-infected in the top panel, and MOI = 0.1 or 1 in the middle or lower panels, respectively) for 24 h. The EV71-induced CPE in the PML^+/+^ and PML^−/−^ cells was examined using microscopy (scale bar: 50 µm). A representative experiment of three experiments is shown. **(B)** The relative VP1 expression in PML^+/+^ or PML^−/−^ cells infected with Gluc-EV71 (MOI = 0.1 or 1) for 24 h is shown. Densitometry was performed, the values from three independent experiments were averaged, and the data are expressed as the fold change in VP1 expression in the PML^−/−^ cells infected at an MOI of 0.1 or 1 or PML^+/+^ cells infected at an MOI of 1 compared with that in the PML^+/+^ cells infected with EV71 at an MOI = 0.1 (Lane 1 is set as 1.00). The VP1 expression in PML^+/+^ cells infected with EV71 at an MOI of 0.1 is assigned a value of 1.00 (**p < 0.01). **(C)** The PML^+/+^ and PML^−/−^ cells were infected with Gluc-EV71 at various MOIs for 24 h, and the cell culture supernatants were measured by performing a luciferase activity assay (Gluc-EV71 infection). The values are the means of three experiments and are presented relative to the level in the mock infection of the PML^+/+^ (white) and PML^−/−^ (red) cells. The data are presented as the mean ± SEM (*n* = 3, **p < 0.01, ***p < 0.001). **(D)** The PML^+/+^ and PML^−/−^ cells were infected with Gluc-EV71 (MOI = 5) for 24 h. The infected cells were identified by staining with an anti-VP1 antibody (green), and DAPI (blue) was used to stain the nuclei. The quantification represented the average of four different fields. Percentage (%) indicates the percentage of infected cells among the total cells. The images were acquired under an Olympus FluoView FV10i confocal microscope (Tokyo, Japan) (scale bar: 50 µm).

This observation was further confirmed in a PML^−/−^ HeLa cell line in which the PML gene is completely knocked out by TALEN (transcription activator-like effector nuclease) technology (19). Gluc-EV71, a reporter virus stably expressing Gaussia luciferase that is capable of efficiently infecting and replicating in various cell types (29), caused more pronounced cytopathic effects in the PML^−/−^ cells than in the PML^+/+^ cells from low to high MOIs (Figure 2A). Similarly, the Western blot analysis showed that VP1 increased by onefold in the PML^−/−^ cells at an MOI of 1 (Figure 2B). To quantify this effect, Gluc-EV71 was used to infect both PML^−/−^ and PML^+/+^ cells, and the luciferase activity was determined. At all MOIs, including the MOI of 0.1, the luciferase activities were significantly higher in the PML^−/−^ cells than those in the PML^+/+^ cells (Figure 2C), suggesting that the knockout of PML renders the cells more susceptible to EV71 infection. The indirect immunofluorescence assay further confirmed that the percentage of infected cells increased by more than twofold in the PML^−/−^ cells compared with that in the PML^+/+^ cells (Figure 2D, 98% positive in the PML^−/−^ cells vs 40% positive in the PML^+/+^ cells). Altogether, PML is a negative regulator of EV71 replication.

### Effect of Various PML Isoforms on EV71 Infection

**Figure 3:**
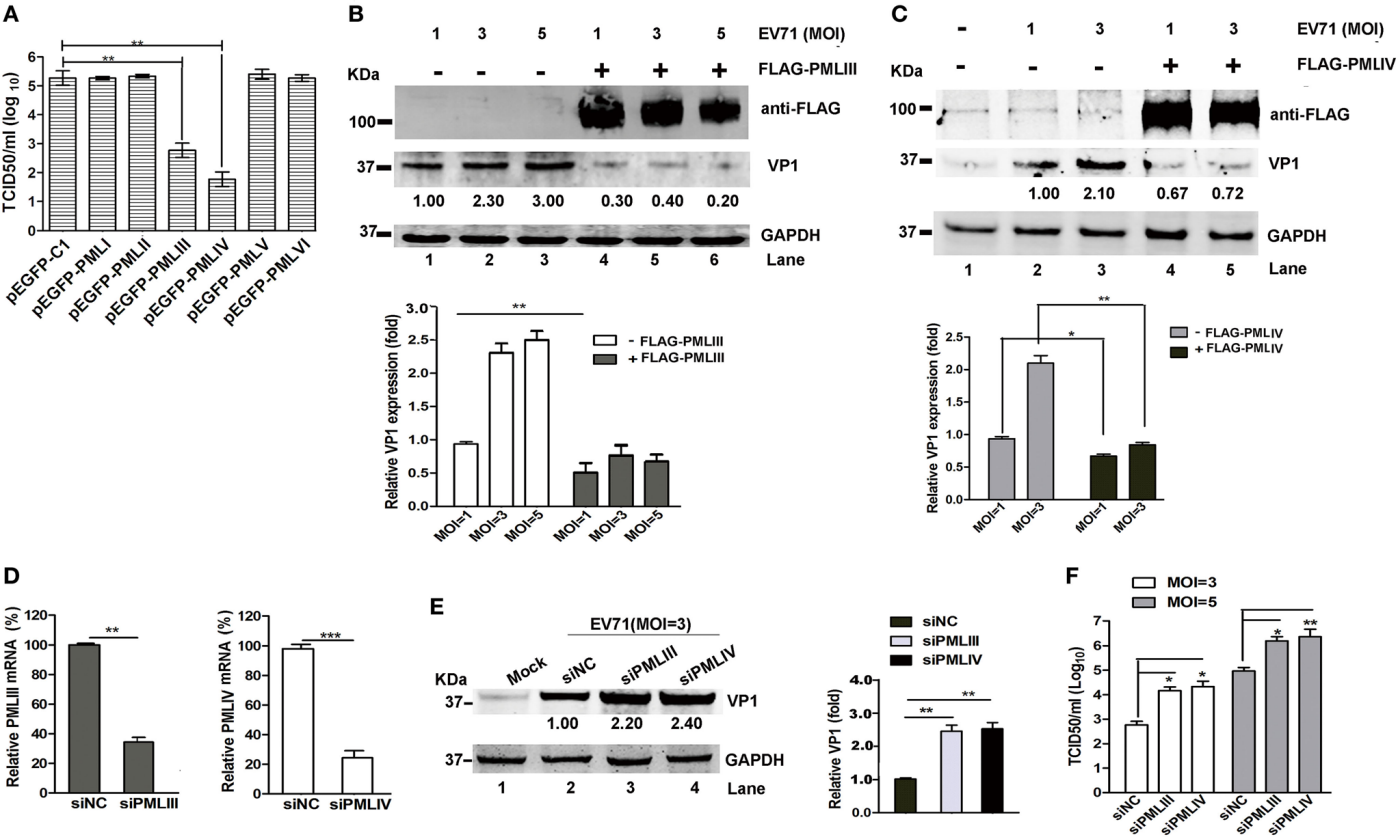
PMLIII and PMLIV isoforms conferred resistance to enterovirus 71 (EV71) infection. **(A)** HeLa cells were transfected with plasmids expressing individual promyelocytic leukemia (PML) isoforms (pEGFP-PMLI to pEGFP-PMLVI) or a control plasmid (pEGFP-C1). At 24 h after transfection, the cells were infected with EV71 at a multiplicity of infection (MOI) of 5. At 24 h post-infection (p.i.), the virus in the culture supernatants was determined by performing a standard plaque assay. The presented values are the means of triplicate determinations and the SEs. The asterisks indicate significant differences (**p < 0.01). **(B,C)** PML^−/−^ HeLa cells were transfected with the plasmids FLAG-PMLIII **(B)**, FLAG-PMLIV **(C)**, or pCDNA3.1 as a control for 24 h and then infected with EV71 at an MOI of 1, 3, or 5 **(B)** or an MOI of 1 or 3 **(C)**. At 24 h p.i., the cell extracts were analyzed by performing a Western blot analysis using an antibody specific for FLAG, VP1, or GAPDH. Densitometry was performed, the values from three independent experiments were averaged, and the data are expressed as the fold change in VP1 expression. The VP1 expression in HeLa cells infected with EV71 at an MOI of 1 is assigned a value of 1.00. **(D)** HeLa cells were transfected with small interfering RNAs (siRNAs) specific for PMLIII or PMLIV or a non-targeting siRNA (siNC) as a control. At 24 h after transfection, the knockdown efficiency of the mRNA of PMLIII or PMLIV was determined by performing qRT-PCR and normalized to the GAPDH mRNA level in the HeLa cells. Data from three independent experiments were averaged and are shown as the fold change in PML expression normalized to siNC. siNC treatment is assigned a value of 100%. **(E)** HeLa cells were transfected with siRNAs specific for PMLIII or PMLIV or a non-targeting siRNA (siNC) for 24 h and then infected with EV71 at an MOI of 3 for 24 h. The cell extracts were determined by performing a Western blot analysis using antibodies specific for VP1 and GAPDH. Densitometry was performed, the values from three independent experiments were averaged, and the data are expressed as the fold change in VP1 expression normalized to siNC. siNC treatment at an MOI of 3 is assigned a value of 1.00 (**p < 0.01). **(F)** HeLa cells were transfected with siRNA specific for PMLIII or PMLIV or a non-targeting siRNA (siNC) for 24 h and then infected with EV71 at an MOI of 3 or 5 for 24 h. The cell culture supernatants were measured by performing a TCID_50_ assay using Vero cells (*p < 0.05, **p < 0.01).

The different PML isoforms have been suggested to play distinct biological roles (30). To determine whether a particular PML isoform is capable of inhibiting EV71 replication, we investigated the effect of overexpressing individual PML isoforms (PMLI- to PMLVI-EGFP) on EV71 infection. Viral production from the infected HeLa cells expressing each PML isoform was analyzed by performing a TCID_50_ assay using Vero cells. The overexpression of isoforms PML-I, PMLII, PMLV, and PMLVI had no effect on the EV71 infection because the viral titers did not differ between cells overexpressing these isoforms and the cells transfected with the empty vector pEGFP-C1 (Figure 3A). By contrast, the viral titers were reduced by 2 and 2.8 logs in cells overexpressing PMLIII and PMLIV, respectively. In addition, to further confirm these observations, HeLa cells were transfected with an empty vector (pEGFP-C1) or plasmids expressing each PML isoform (PMLI–VI) followed by infection with EV71 (MOI = 5) for 24 h. The expression levels of the different PML isoforms were detected using an anti-FLAG antibody (Figure S1 in Supplementary Material), and the result is consistent with the previous study (19). The Western blot analysis of extracts from these cells infected at an MOI of 5 confirmed the above-mentioned result that PMLIII and PMLIV, but not the other isoforms, inhibited EV71 VP1 expression (Figure S1 in Supplementary Material). The double immunofluorescence staining of PML and the EV71 VP1 antigen revealed that the PMLIII- and PMLIV-stained cells were distinct from the VP1-stained cells, suggesting that PMLIII and PMLIV inhibited VP1 expression (Figure S1 in Supplementary Material). This result was corroborated by the reduction of VP1, as shown by Western blot analysis of the cell lysates. The overexpression of isoform PMLIII (Figure 3B, Lanes 4, 5, and 6) inhibited VP1 expression in cells infected at different MOIs (Figure 3B, Lanes 1, 2, and 3). Similar result was observed for isoform PMLIV (Figure 3C). These observations are consistent with the reduction in the viral titers (Figure 3A). To further evaluate the inhibitory activity of PMLIII and PMLIV in EV71 replication, we inhibited the cellular expression of either PMLIII or PMLIV using specific siRNAs and analyzed the EV71 infection by measuring VP1 expression. The expression of both PMLIII and PMLIV was effectively suppressed by the respective siRNAs as shown in Figure 3D. VP1 expression increased accordingly in the cells with suppressed PMLIII and PMLIV expression, suggesting that cellular PMLIII and PMLIV inhibit viral replication (Figure 3E, Lanes 3 and 4 compared with Lane 2). Consistently, the progeny viral titers increased by more than one log in the cells treated with siPMLIII or siPMLIV (Figure 3F).

### PML Repressed the EV71 Infection Partially by Inhibiting Autophagy, and PML Deficiency Triggered Autophagy Formation

**Figure 4:**
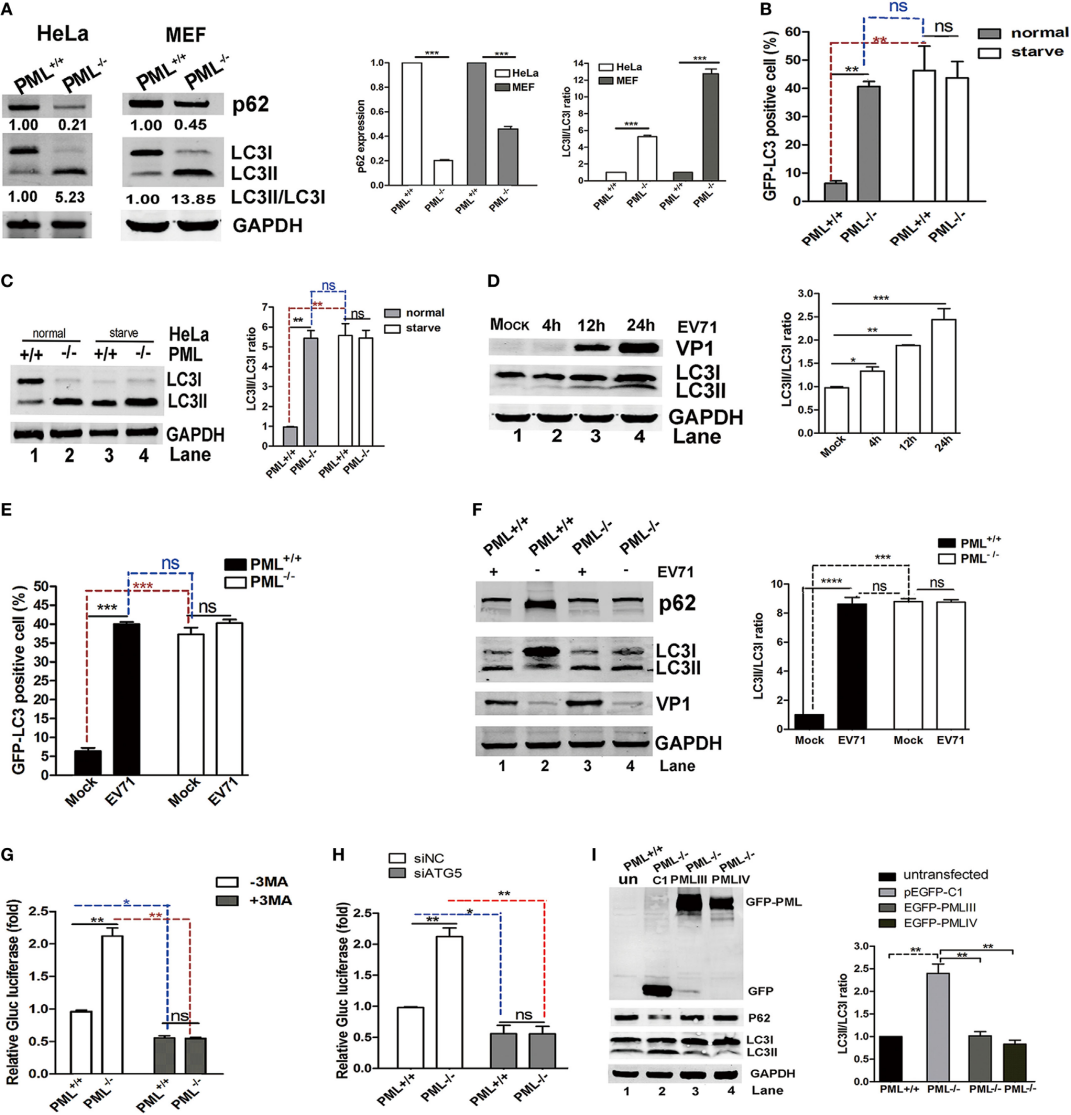
Promyelocytic leukemia (PML) downregulation triggered autophagy, and PML regulated enterovirus 71 (EV71) infection by inhibiting autophagy. **(A)** The conversion of endogenous LC3-I to LC3-II and p62 degradation were detected in PML^+/+^ and PML^−/−^ HeLa cells or mouse embryonic fibroblast (MEF) cells. The protein levels of LC3-I, LC3-II, and p62 in the same number of PML^+/+^ and PML^−/−^ cells were measured by performing a Western blot analysis. The ratio of LC3-II/LC3-I (right panel) and the relative expression of p62 (middle panel) represent the relative density of the bands compared with that of the corresponding control normalized to GAPDH. The value of the band(s) of the PML^+/+^ HeLa or MEF cells is set as 1.00 (100%). The data are expressed as the mean ± SEM of three separate experiments. **(B)** Percentages of GFP-LC3 puncta-positive cells in PML^+/+^ and PML^−/−^ HeLa cells transfected with the GFP-LC3 plasmid under normal conditions (the left panel) and after starvation (the right panel). Cells were transfected with the GFP-LC3 plasmid for 12 h, followed by the treatment with normal conditions or serum deprivation for 2 h. The percentage of cells with punctate GFP-LC3 was expressed as cells with two or more puncta vs total GFP-positive cells. The samples were examined under a fluorescence microscope, and the images were analyzed under an Olympus FluoView FV10i confocal microscope (Tokyo, Japan). A minimum of 50 cells from each treatment was counted in four different fields, and the percentage of cells with punctate GFP-LC3 is shown in the right panel. Bars indicate SEM (**p < 0.01, ***p < 0.01, *n* = 4). **(C)** The protein levels of LC3-I and LC3-II in PML^+/+^ and PML^−/−^ HeLa cells under normal or serum deprivation conditions at 2 h p.i. were measured by performing a Western blot analysis. The ratio of LC3-II/LC3-I represents the relative density of the bands compared with that of the corresponding control normalized to GAPDH. The ratio of LC3-II/LC3-I in PML^+/+^ HeLa cells that received the mock treatment is set as 1.00 (100%). Three independent experiments were performed, and a representative image is shown; the data are expressed as the mean ± SEM of three separate experiments. **(D)** HeLa cells were infected with EV71 at a multiplicity of infection (MOI) of 5 at the indicated time point, and the protein levels of LC3-I, LC3-II, and VP1 were measured by performing a Western blot analysis. The ratio of LC3-II/LC3-I represents the relative density of the bands compared with that of the corresponding control normalized to GAPDH. The ratio of LC3-II/LC3-I in HeLa cells that received the mock treatment is set as 1.00 (100%). Three independent experiments were performed, and representative data are shown. **(E)** Percentages of GFP-LC3 puncta-positive cells in PML^+/+^ and PML^−/−^ HeLa cells transfected with the GFP-LC3 plasmid either infected or mock-infected with EV71 (MOI = 5) at 12 h p.i. The cells were stained with anti-EV71 mAb and Alexa Fluor 546-conjugated anti-mouse IgG antibody. Images were acquired under an Olympus FluoView FV10i confocal microscope (Tokyo, Japan). DAPI is shown in blue, and VP1 is shown in red. Bars indicate SEM. *n* = 4 (ns indicates no significance, **p < 0.01, ***p < 0.001). A minimum of 50 cells from each treatment was counted in four different fields, and the percentage of cells with punctate GFP-LC3 is shown. **(F)** The protein levels of LC3-I, LC3-II, and p62 in PML^+/+^ and PML^−/−^ HeLa cells either mock-infected or infected with EV71 (MOI = 3) at 12 h p.i. were measured by performing a Western blot analysis. The ratio of LC3-II/LC3-I represents the relative density of the bands compared with that of the corresponding control normalized to GAPDH. The value of LC3-II/LC3-I in PML^+/+^ HeLa cells that received the mock treatment is set as 1.00 (100%). Three independent experiments were performed, and representative data are shown. **(G)** PML^+/+^ and PML^−/−^ HeLa cells were infected with Gluc-EV71 (MOI = 5) for 48 h in the presence or absence of 3-methyladenine (3-MA) (250 µM). The virus supernatant was examined by measuring the luciferase activity. The expression of luciferase activity represented the viral replication in comparison to the control (without 3-MA) in virus-infected PML^+/+^ cells. The value of the luciferase activity in infected PML^+/+^ cells without 3-MA treatment is set as 1.00 (100%). Bars indicate SEM (*p < 0.05, **p < 0.01, *n* = 3). **(H)** PML^+/+^ or PML^−/−^ HeLa cells were transfected with siATG5 or non-specific small interfering RNA (siNC) for 24 h and then infected with Gluc-EV71 at an MOI of 5 for 48 h. The virus supernatant was examined by measuring the luciferase activity. The expression of luciferase activity represents the viral replication and was compared with that in the control and virus-infected PML^+/+^ cells. The value of the luciferase activity in infected PML^+/+^ cells that received the siNC treatment is set as 1.00 (100%). Bars indicate SEM (*p < 0.05, **p < 0.01, *n* = 3). **(I)** PML^−/−^ HeLa cells in 12-well plates were transfected with an equal quantity of pEGFP-C1, pEGFP-PMLIII, or pEGFP-PMLIV for 24 h. PML^−/−^ HeLa cells transfected with pEGFP-C1 were used as a control. The relative expression of LC3-II/LC3-I represents the relative density of the band compared with that of the corresponding control normalized to GAPDH. The ratio of LC3-II/LC3-I in PML^+/+^ HeLa cells that received the mock treatment is set as 1.00 (100%). Three independent experiments were performed, and representative data are shown.

Viral infections result in autophagy, which was first reported in the 1960s (31). Effect of autophagy on viral infections has become increasingly appreciated (32). In recent studies, autophagy was shown to facilitate or restrict viral replication in a virus-specific manner (33–35). Virus-associated autophagy acts both as an antiviral and proviral mechanisms and the biological roles of autophagy depend on the virus, cell type, and cellular environment (32). Virus-induced autophagy facilitated EV71 replication both *in vitro* and *in vivo* (26, 36, 37). Therefore, we tested whether PML regulates EV71 replication *via* autophagy. To determine the possible involvement of PML in the autophagic process in HeLa and MEF cells, we examined the level of the autophagy marker LC3 in PML^+/+^ and PML^−/−^ HeLa and MEF cells, respectively. The redistribution of LC3 to autophagosomes is usually accompanied by its lipidation, causing an increase in its electrophoretic mobility and, hence, a shift from LC3-I to LC3-II (38), which is widely used as an indicator of autophagosome formation. A decrease in p62 expression and a marked increase in LC3-II expression were observed in both the PML^−/−^ HeLa and PML^−/−^ MEF cells (Figure 4A). To further confirm the possible involvement of PML in the autophagic process, we monitored the autophagosome levels in PML^+/+^ and PML^−/−^ HeLa cells under normal conditions and after serum deprivation. The PML^+/+^ and PML^−/−^ HeLa cells were transfected with a green-fluorescent LC3 plasmid (pEGFP-LC3), and 24 h after transfection, the cells were cultured under serum-deprived or normal conditions. The autophagosomes were evaluated by performing an immunofluorescence assay and quantifying the GFP-LC3-positive dots. In contrast to the diffused pattern of GFP-LC3 observed in PML^+/+^ cells under normal conditions, the GFP-LC3-positive dots were clustered and more abundant in the PML^−/−^ cells under normal conditions (Figure 4B). The serum deprivation treatment triggered a significant increase in the number of GFP-LC3 puncta-positive cells in the PML^+/+^ cells but did not cause significant changes in the number and distribution pattern in the PML^−/−^ cells. Based on the quantification of the LC3-positive cells, the level of autophagosome formation in the PML^+/+^ cells under the serum deprivation condition was similar to that in the PML^−/−^ cells under normal conditions, suggesting that depletion of PML triggers autophagy. Our observation is consistent with an earlier study showing that autophagosome formation did not significantly change after starvation and exposure to other pro-autophagic stimuli in PML^−/−^ MEF cells (39). In the analysis of the LC3-I to LC3-II conversion in our study, the PML^−/−^ HeLa cells had significantly higher LC3-II/I ratios than the PML^+/+^ HeLa cells under the normal conditions, while the PML^+/+^ and PML^−/−^ cells had similarly higher LC3-II/I ratios under the serum-deprived conditions (Figure 4C). Interestingly, the level of LC3-II in the PML^+/+^ cells under the serum-deprived conditions was the same as that in the PML^−/−^ cells under both the normal and serum-deprived conditions. Therefore, the depletion of PML triggers autophagy, and further stress stimulation (serum deprivation) does not aggravate the autophagosome formation, suggesting that PML plays critical roles in regulating autophagosome formation in HeLa cells.

In addition, autophagy was induced in the HeLa cells during the EV71 infection, and the LC3-II/LC3-I ratio gradually increased as the infection proceeded from 4 to 24 h p.i. as shown in Figure 4D. Although both the numbers of autophagosomes (Figure 4E) and LC3-II/I ratio (Figure 4F) were higher in the PML^−/−^ cells than those in the PML^+/+^ cells under the mock infection, the numbers of autophagosomes and LC3-II/I ratio were elevated in PML^+/+^ but not in PML^−/−^ cells following infection with EV71 (Figures 4E,F). The level of autophagosomes (Figure 4E) and LC3-II/I ratio (Figure 4F) in the mock-infected and EV71-infected PML^−/−^ cells were similar, suggesting that autophagy was mainly induced by PML knockout and not further induced by the viral infection. We also assayed the level of the autophagic substrate p62/SQSTM1 in PML^+/+^ and PML^−/−^ cells infected or mock-infected with EV71 and found that level of p62 protein was reduced in the mock-infected PML^−/−^ cells as compared with that in the mock-infected PML^+/+^ cells (Figure 4F, Lane 4 compared with Lane 2), suggesting the autophagic degradation of p62. The EV71 infection induced a marked reduction in p62 in the PML^+/+^ cells (Figure 4F, Lane 1 compared with Lane 2) but had no effect on the PML^−/−^ cells (Figure 4F, Lane 3 compared with Lane 4). Thus, PML knockout resulted in higher autophagy, which was not affected by the viral infection. The level of autophagosome formation in the infected or serum-deprived PML^+/+^ cells was similar to that in the PML^−/−^ cells under normal conditions, and further infection or nutrition depletion in the PML^−/−^ cells did not further elevate autophagy, suggesting that the depletion of PML alone is sufficient to induce autophagosome formation. Altogether, we propose that PML deficiency triggered autophagy in HeLa cells and EV71-induced autophagy requires the presence of PML, implicating that PML plays critical roles in virus-induced autophagic flux. We hypothesize that using 3-MA, an inhibitor of the Beclin-1-dependent class III phosphoinositide 3-kinase, to block the autophagosome formation could reduce EV71 replication. As shown in Figure 4G, the Gluc-EV71 luciferase activity increased by more than twofold in the PML^−/−^ cells compared with that in the PML^+/+^ cells (open columns). However, the 3-MA treatment significantly reduced the Gluc-EV71 luciferase activity to a similar level in both the PML^+/+^ and PML^−/−^ cells, suggesting that the PML inhibition of EV71 replication is mediated by a negative regulation of autophagy; however, we cannot absolutely rule out a direct role of PML or PML-NBs in EV71 replication. To further substantiate this observation, we knocked down the key autophagy-associated protein ATG5 using specific siRNA to block the generation of autophagosomes and analyzed the viral replication. Compared to the negative control siNC, siATG5 reduced the intracellular ATG5 protein level by 95% (Figure S2 in Supplementary Material, right bottom panel). The ATG5-knockdown WT HeLa cells failed to show an increase in LC3-II upon EV71 infection (Figure S3 in Supplementary Material, left bottom panel), and a drastically reduced VP1 expression was observed (Figure S3 in Supplementary Material, upper panel). The siATG5 treatment in the PML^+/+^ and PML^−/−^ HeLa cells reduced the Gluc-EV71 luciferase activity to a similar level as the siNC treatment, suggesting that PML repressed EV71 replication by inhibiting autophagy (Figure 4H). To further illustrate the effect of PML on autophagy, we reintroduced PMLIII or PMLIV into PML^−/−^ cells by transfecting the cells with the plasmids PMLIII or PMLIV or the control plasmid pEGFP-C1 and analyzed the LC3-II expression. LC3-II was reduced in both the PMLIII-EGFP- and PMLIV-EGFP-transfected cells compared with that in the p-EGFP-C1-transfected cells (Figure 4I, Lanes 3 and 4 compared with Lane 2), suggesting that the expression of either isoform, i.e., PMLIII or PMLIV, inhibited autophagy in the PML^−/−^ cells. Altogether, isoforms PMLIII and PMLIV inhibit EV71 replication by inhibiting autophagy.

### Antiviral Activity of IFN-β Required the Presence of PML

**Figure 5:**
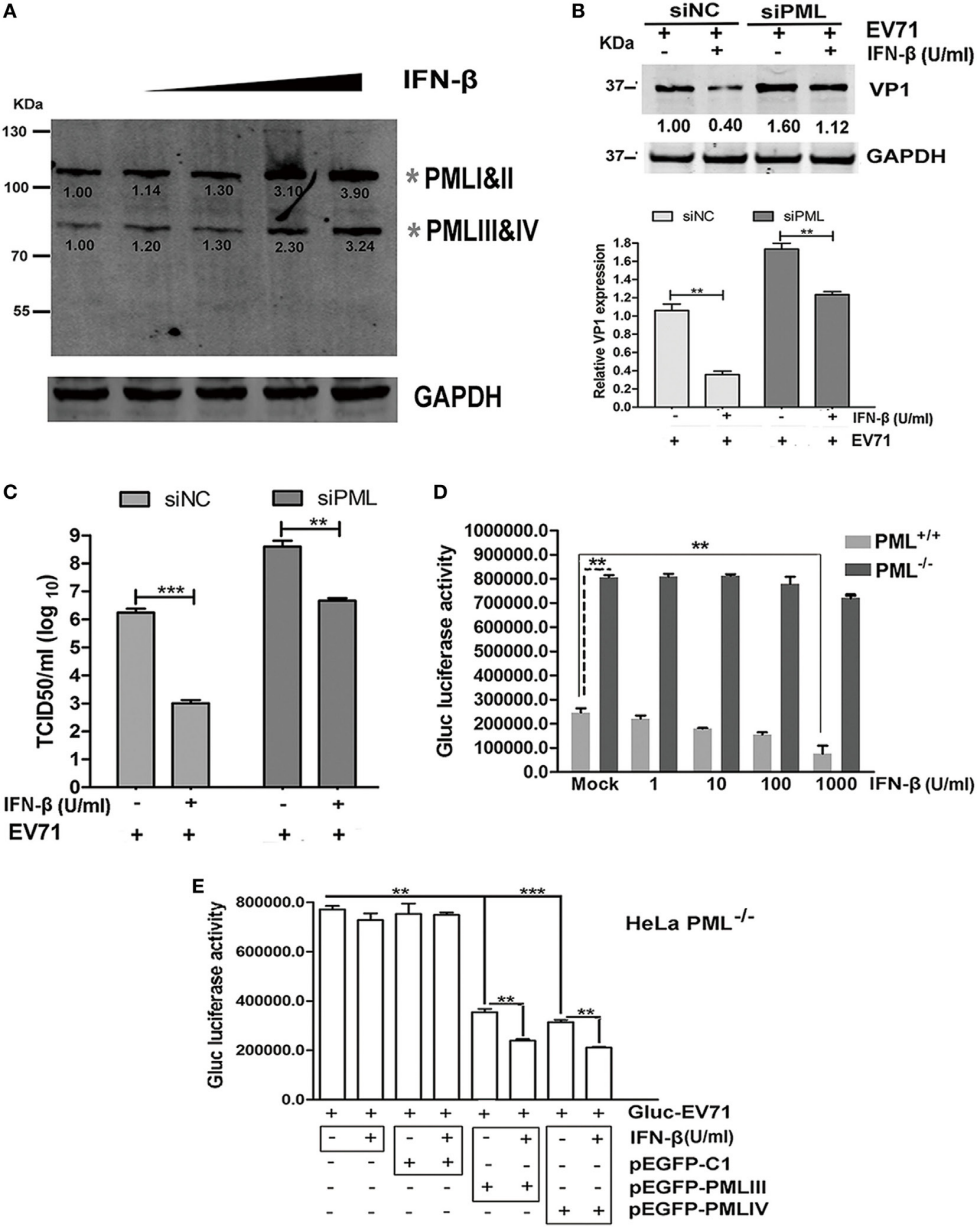
Antiviral activity of interferon (IFN)-β required the presence of promyelocytic leukemia (PML). **(A)** HeLa cells were treated with IFN-β at various concentrations (10–1,000 U/ml) for 24 h, and the endogenous PML isoforms were detected by performing a Western blot analysis. The gray asterisks indicate PML isoforms I and II or PMLIII and IV. **(B,C)** HeLa cells were transfected with siNC or siPML for 24 h, and the siNC- or siPML-treated cells were treated with or without IFN-β (1 × 10^3^ U/ml) for 12 h and then infected with Gluc-enterovirus 71 (EV71) at an multiplicity of infection (MOI) of 5 for 24 h. The cell lysates were analyzed by performing a Western blot analysis using antibodies against VP1 and GAPDH. The density of the bands was scanned by performing densitometry and normalized to that of GAPDH. The VP1 value in the cells treated with siNC and infected with EV71 without IFN-β treatment is set as 1.00 **(B)**. The culture supernatants were measured by performing a TCID_50_ assay using Vero cells **(C)**. Three independent experiments were performed, and representative data are shown. **(D)** PML^+/+^ and PML^−/−^ cells were treated with IFN-β at various concentrations for 24 h and mock-infected or infected with Gluc-EV71 (MOI = 1) for 48 h, and the virus supernatants were measured by performing a luciferase activity assay. The presented values are the mean of triplicate determinations and SEM. Three independent experiments were performed, and representative data are shown. Asterisks indicate significant differences (**p < 0.01). **(E)** PML^+/+^ and PML^−/−^ cells were treated with IFN-β at 1 × 10^3^ U/ml for 24 h and transfected with pEGFP-C1, pEGFP-PMLIII, or pEGFP-PMLIV for 24 h. Then, the cells were infected with Gluc-EV71 at an MOI of 1 for 48 h, and the culture supernatants were measured by performing a luciferase activity assay. The presented values are the means of triplicate determinations and SEM. Three independent experiments were performed, and representative data are shown. The asterisks indicate significant differences (***p < 0.001, **p < 0.01).

The effect of exogenous IFN-β on PML expression was investigated by performing a Western blot analysis using an antibody specific for all isoforms. IFN-β exhibited differential effects on the expression of the PML isoforms, and an elevation in isoforms PMLIII and IV was observed in response to increasing IFN-β (Figure 5A). To investigate the antiviral effect of IFN-β, we compared the antiviral activity of IFN-β in siPML-treated cells with that in siNC-treated HeLa cells infected with EV71 by performing a Western blot analysis of VP1 expression or virus titer determination. The siNC- and siPML-treated HeLa cells were stimulated in the presence or absence of IFN-β for 24 h followed by infection with Gluc-EV71 (MOI = 5). The antiviral effect of IFN-β was lower in the siPML-treated HeLa cells than that in the siNC-treated HeLa cells (Figures 5B,C). To further confirm the role of PML in the antiviral activity of IFN-β, we evaluated the effect of IFN-β on EV71 replication in the PML^+/+^ and PML^−/−^ HeLa cells. Cells treated with or without various concentrations of IFN-β were infected with Gluc-EV71 (MOI = 1) and the viral replication was analyzed by measuring the luciferase activity at 48 h p.i. In the PML^+/+^ cells, IFN-β dose dependently reduced the luciferase activity; however, the luciferase activity in the PML^−/−^ cells was not significantly affected even at the highest IFN-β concentration of 1 × 10^3^ U/ml (Figure 5D), suggesting that the antiviral activity of IFN-β requires the presence of PML.

To further confirm the roles of PML in the mediation of an IFN-induced antiviral state, we transfected the PML^−/−^ cells with PMLIII, PMLIV, or an empty vector as a negative control in the presence of absence of IFN-β (1 × 10^3^ U/ml) and infected the cells with Gluc-EV71 (MOI = 1) for 48 h. The inhibition of virus replication by either isoform PMLIII or PMLIV was significantly enhanced by the presence of IFN-β (Figure 5E). In the absence of either the PMLIII or PMLIV isoform, the viral replication was not affected by IFN-β. The control plasmid (pEGFP-C1) had no effect on the antiviral activity of IFN-β and did not impact the viral replication (Figure 5E). Thus, the antiviral activity of IFN-β requires the presence of either PMLIII or PMLIV, and isoforms PMLIII and PMLIV likely mediate an IFN-β-induced antiviral state against EV71 replication.

### EV71 Infection Disrupted PML-NB Formation and Induced a Late Reduction in the PML Level

**Figure 6:**
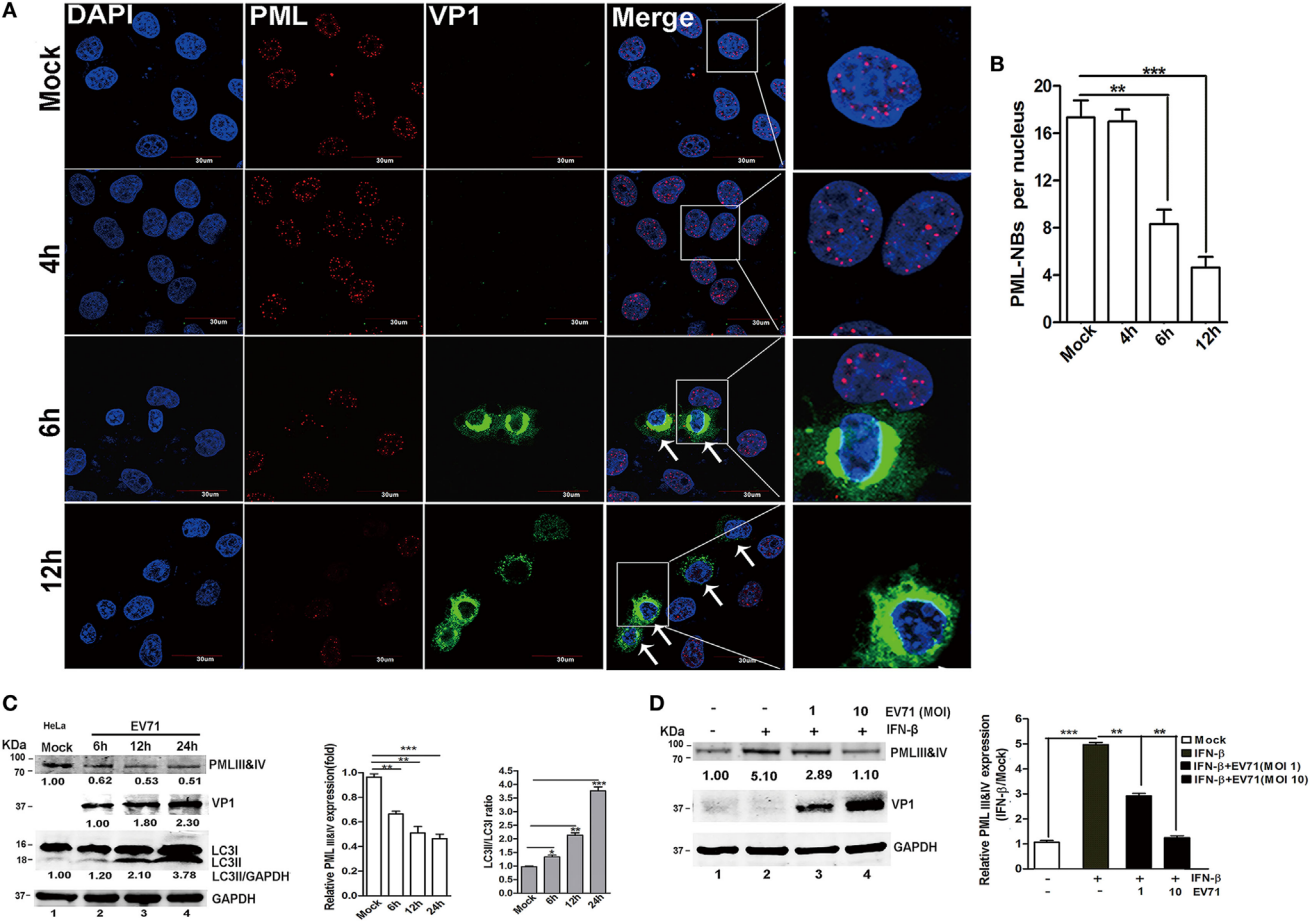
Enterovirus 71 (EV71) infection downregulated promyelocytic leukemia protein-nuclear bodies (PML-NBs) and resulted in a reduction in the promyelocytic leukemia (PML) protein level. **(A)** Alterations in PML-NBs in EV71-infected HeLa cells. HeLa cells grown on 12-well slides were mock-infected (upper panel) or infected with EV71 [multiplicity of infection (MOI) = 5]. At the indicated time points, the cells were fixed and stained with polyclonal antibodies against PML (red) and VP1 (green), and DAPI is shown in blue; the images were acquired under a confocal microscope. White arrows indicate the EV71-infected HeLa cells (PML-NBs were absent from the nuclei) (scale bar: 30 µm). The images are representative of three independent experiments. **(B)** A minimum of 50 cells was counted for the number of PML-NBs, and the mean number of PML-NBs per nucleus in the uninfected and infected cells in each group is presented. The presented values are the mean ± SEM (**p < 0.01, ***p < 0.001). **(C,D)** EV71 infection led to a decrease in the endogenous PMLIII and IV isoforms in cells treated with or without interferon (IFN)-β. **(C)** EV71 infection led to a decrease in endogenous PMLIII and IV isoforms at a later stage in the HeLa cells. Cells were mock-infected or infected with EV71 at an MOI of 5, and the cell lysates were analyzed at 6, 12, or 24 h post-infection (p.i.) by performing a Western blot analysis using antibodies against PML, LC3, VP1, or GAPDH. The relative expression of PMLIII and IV represents the density of the band compared with that of the corresponding control normalized to GAPDH. The value of the PMLIII and IV isoforms in the mock-infected cells (Lane 1) is set as 1.00 (100%). The ratio of LC3-II/LC3-I represents the relative density of the bands compared with that of the corresponding control normalized to GAPDH. The value of LC3-II/LC3-I in the HeLa cells that received the mock treatment is set as 1.00 (100%). Three independent experiments were performed, and representative data are shown. **(D)** EV71 infection reduced PMLIII and IV expression in IFN-β-treated HeLa cells. HeLa cells were treated with or without IFN-β (1,000 U/ml) for 24 h and then washed with fresh medium before being infected with EV71 at an MOI of 1 or 10. The cell lysates were analyzed by performing a Western blot analysis at 24 h p.i. using antibodies against PML, VP1, or GAPDH. The relative expression of PMLIII and IV represents the density of the band compared with that of the corresponding control normalized to GAPDH. The density value of PMLIII and IV in the mock-infected cells (Lane 1) is set as 1.00 (100%). All experiments were performed three times, and representative results are shown.

The PML-NBs decreased in the nuclei of the EV71-infected HeLa cells. Upon infection, a significant reduction in PML-NBs was observed as early as 6 h p.i., and the PML-NBs were reduced even further at 12 h p.i. (Figure 6A, as indicted by the white arrows). To quantify the reduction in the PML-NBs in the infected cells, the PML-NB puncta were counted in 30 nuclei. As shown in Figure 6B, the EV71 infection resulted in a fourfold reduction in PML-NBs in the HeLa cells at 12 h p.i. compared with that in the mock-infected cells. However, the PML-NB size was the same in the mock-infected and EV71-infected cells. Thus, the EV71 infection altered the numbers of PML-NBs without affecting the PML-NB size.

To determine whether the *de novo* expression of the PML protein (isoforms PMLIII and/or PMLIV) was affected, cell lysates from HeLa cells were infected with EV71 for various durations, and the PMLIII and/or PMLIV expression levels were analyzed by Western blotting (Figure 6C). The EV71 infection led to a significant reduction in the PMLIII and/or PMLIV protein levels as early as 6 h p.i. (Figure 6C, Lane 2 compared with Lane 1, PMLIII of 78 kDa and PMLIV of 78 kDa co-migrating as a single band on SDS-PAGE) in HeLa cells. IFNs directly induce PML gene expression, resulting in an increase in several PML isoforms (2, 6) and their expression is critical for host antiviral defense (40). Therefore, we investigated if EV71 infection could downregulate the endogenous PMLIII and/or PMLIV upregulated by IFN-β pre-treatment. HeLa cells were pre-treated with IFN-β for 24 h following with or without EV71 infection at different MOIs for 24 h. In the mock-infected cells, IFN-β dramatically upregulated the PML gene, resulting in an increase in PMLIII and/or PMLIV, and the IFN-β-induced upregulation of PMLIII and/or PMLIV were partially mitigated by the EV71 infection as shown in Figure 6D. At an MOI of 10, the IFN-β-upregulated PMLIII and/or PMLIV were almost reduced to levels similar to those without the IFN-β treatment (Figure 6D, Lane 4 compared with Lane 1). The result in 293T cells was consistent with the result in the HeLa cells (data not shown). The IFN-β treatment also upregulated the expression of other PML isoforms, particularly PMLI and II, that were similarly mitigated by the EV71 infection (data not shown). To determine whether the EV71-induced downregulation of PML was mediated by the inhibition of PML mRNA synthesis, HeLa cells were infected with EV71 (MOI = 2), and the total RNA was isolated for a qPCR analysis. However, the PML mRNA levels did not differ between the mock-infected and infected cells during the entire duration of infection (data not shown). Thus, the reduction in the PML protein is a consequence of its degradation.

### EV71-Induced Degradation of PML Is Independent of the Proteasome Pathway and Mediated by 3C^pro^, but Not 2A^pro^

**Figure 7:**
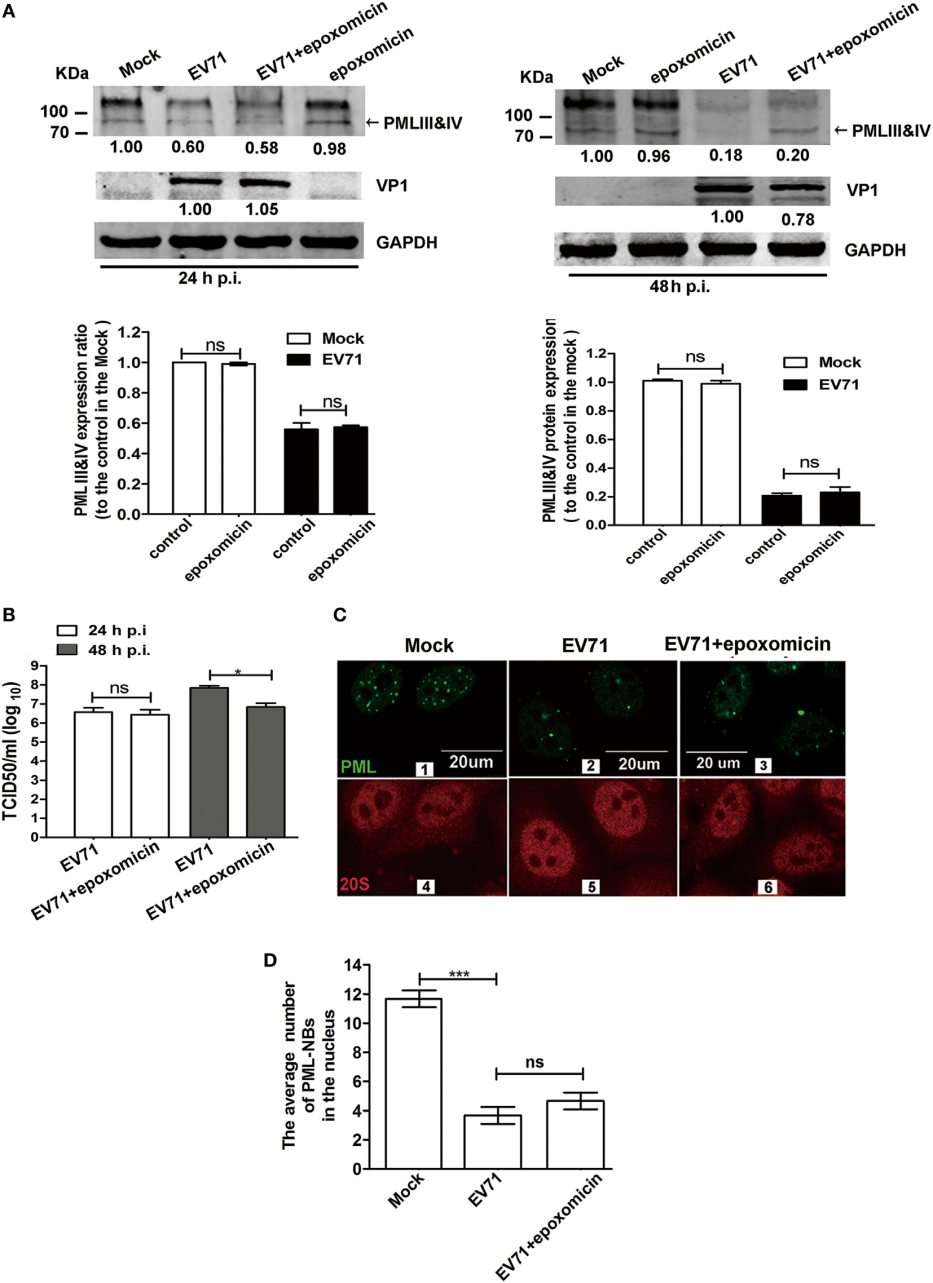
Enterovirus 71 (EV71)-induced promyelocytic leukemia (PML) degradation was not mediated by the proteasome pathway. **(A)** Epoxomicin did not inhibit the EV71-induced PMLIII and IV degradation. Cell lysates were prepared from 293T cells infected or mock-infected with EV71 at a multiplicity of infection (MOI) of 5 for 24 or 48 h in the presence or absence of epoxomicin (1 µM). At 24 h post-infection (p.i.) (left panels) or 48 h p.i. (right panels), 60 μg of protein extracted from each treatment were separated on SDS-PAGE and analyzed by performing a Western blot analysis using antibodies specific for PMLIII and IV (top panels), VP1 (middle panels), and GAPDH, which served as an internal loading control (bottom panels). PMLIII and IV were quantified by performing densitometry and are presented relative to the mock infection, which was set as 1.0. VP1 was quantified and is presented relative to that in the EV71-infected 293T cells at 24 h p.i. (left panels) or 48 h p.i. (right panels). The density value of VP1 without epoxomicin is set as 1.0. The experiment was performed three times, and representative results are shown. **(B)** The progeny virus in the culture supernatants of 293T cells infected with EV71 at an MOI of 5 in the presence or absence of epoxomicin at 24 or 48 h p.i. was measured by performing a TCID_50_ assay using Vero cells (*p < 0.05, **p < 0.01). **(C)** Confocal microscopic analysis of promyelocytic leukemia protein-nuclear bodies (PML-NBs) and the proteasome 20S in EV71-infected HeLa cells. HeLa cells were infected or mock-infected with EV71 at an MOI of 5 for 24 h. Double immunofluorescence staining was performed using a polyclonal anti-PML antibody and Alexa 488 as a secondary antibody or a rabbit anti-20S and Alexa 594 as a secondary antibody (scale bar: 20 µm). The images are representative of three independent experiments. **(D)** The total number of PML-NBs in each nucleus was counted in three views under a confocal microscope, and the average number of PML-NBs per nucleus was calculated. In total, 30 cells were counted. The presented values are the means of triplicate determinations and SEM. Asterisks indicate significant differences (***p < 0.001), and *ns* indicates no significant difference.

Earlier studies revealed that HSV-1 and EMCV infections mediated PML degradation in a proteasome-dependent manner (7, 14, 41). The addition of epoxomicin or MG132 abrogated the HSV-1-induced PML degradation in cells overexpressing PML and cells treated with IFN-α. PML transferring from the nucleoplasm to the nuclear matrix induced by EMCV infection led to a bigger size of PML-NBs (41). The proteasome component 20S co-localized with PML within PML-NBs, following by PML degradation in a proteasome- and SUMO-dependent manner (41). To determine whether the PMLIII and IV degradation is dependent on the proteasome pathway, we infected 293T cells with EV71 in the presence or absence of epoxomicin (1 µM) for various time durations (24 and 48 h p.i.) and conducted a Western blot analysis of PML expression. Epoxomicin could not prevent the EV71-induced PML degradation at 24 and 48 h p.i. (Figure 7A). We also evaluated the effect of epoxomicin on VP1 expression in the cell extracts and measured the viral titers in the same culture supernatants; epoxomicin did not affect the virus replication (Figure 7A, up left panel) and the virus titer (Figure 7B, open column) at 24 h p.i. However, epoxomicin inhibited the EV71 VP1 expression (Figure 7A, upper right panel) and reduced the virus titer by one log at 48 h p.i. (Figure 7B, gray column). Although epoxomicin inhibited EV71 replication, it did not prevent PML from degradation in the EV71-infected 293T cells at 48 h p.i. The MG132 treatment also failed to prevent the PML degradation in the EV71-infected 293T cells (data not show). Thus, the PML degradation is independent of the proteasome pathway.

We further investigated whether epoxomicin could prevent the PML-NB disruption in the EV71-infected HeLa cells. An immunofluorescence analysis was performed to examine the number of PML-NBs and the localization of the 20S proteasome during EV71 infection using antibodies specific for PML and proteasome 20S in the IFN-β-treated HeLa cells. In the mock-infected cells, the proteasome 20S located in both the cytoplasm and the nucleus (Figure 7C 4). However, no difference was observed in the proteasome 20S distribution between the mock-infected and infected cells (Figure 7C 4 and 5). To quantify the average number of PML-NBs in the nuclei of the mock-infected and infected cells, the PML-NB puncta were counted in 30 nuclei from each group. Epoxomicin did not prevent the PML-NB disruption induced by the viral infection (Figure 7C 2 vs 3; Figure 7D). Taken together, these findings suggest that EV71-induced PML-NB degradation is not mediated by the proteasome pathway.

**Figure 8:**
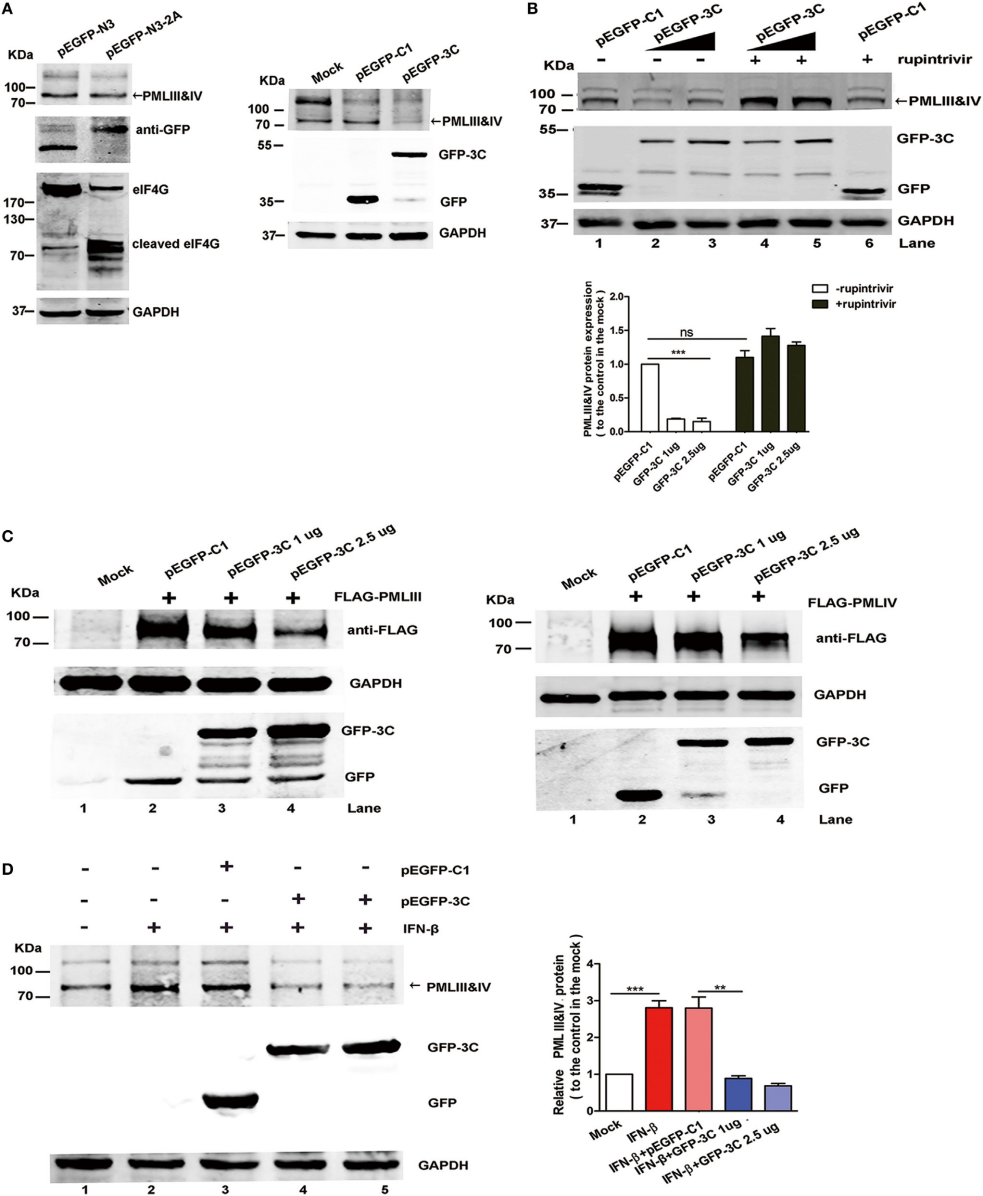
Viral 3C^pro^, but not 2A^pro^, induced promyelocytic leukemia (PML) degradation. **(A,B)** The endogenous PMLIII and IV in the HeLa cells were degraded by viral 3C^pro^ but not 2A^pro^. **(A)** HeLa cells transfected with the plasmids pEGFP-N3 (control) or pEGFP-N3-2A (left panel) and pEGFP-C1 or pEGFP-3C (right panel) for 24 h were lysed and analyzed by performing a Western blot analysis using antibodies against PML, GFP, eukaryotic translation initiation factor 4G (eIF4G), and GAPDH. **(B)** Effect of 3C^pro^ inhibitor rupintrivir on PMLIII and IV degradation. HeLa cells were transfected with control plasmid pEGFP-C1 or increasing amounts of pEGFP-3C. At 24 h post transfection, the cells were incubated with rupintrivir (2 µM) for 24 h. Then, the cell lysates were processed for a Western blot analysis. The density of the bands was scanned by performing densitometry and expressed relative to that of PMLIII and IV without 3C (pEGFP-C1) and rupintrivir. The density value of PMLIII and IV in the mock-treated cells (Lane 1) was set as 1.00 (100%). All experiments were performed three times, and representative results are shown. **(C)** 293T cells were co-transfected with the plasmids pEGFP-C1 (2.5 µg) or pEGFP-3C (1.0, or 2.5 µg) with PMLIII (left panel) or PMLIV (right panel) for 24 h, respectively. The cells were lysed and analyzed by performing a Western blot analysis using antibodies against FLAG, GFP, and GAPDH. **(D)** The 3C^pro^ expression induced endogenous PMLIII and IV protein degradation in the presence of interferon (IFN)-β (1,000 U/ml) in the 293T cells. The 293T cells were exposed to IFN-β (1,000 U/ml) for 24 h, and the IFN-β was removed using fresh medium before the transfection with the plasmid pEGFP-3C or pEGFP-C1 (control) for 24 h. The whole cell lysates were analyzed by performing a Western blot analysis using a rabbit polyclonal antibody against PML or antibodies against GFP and GAPDH. The relative expression of PMLIII and IV represents the density of the bands compared with that of the corresponding control normalized to GAPDH. The density value of PMLIII and IV in the mock-treated cells (Lane 1) is set as 1.00 (100%). All experiments were performed three times, and representative results are shown.

2A^pro^ and 3C^pro^, two EV71 encoded viral proteases, are important for processing viral protein precursors and have been reported to cleave a large number of cellular molecules that affect the host cellular functions (42–45). Since isoforms PMLIII and IV were degraded upon the EV71 infection, we investigated the roles of these viral proteases in the degradation. We examined whether 2A^pro^ and 3C^pro^ had cleavage activity against PML by transfecting a 2A^pro^- or 3C^pro^-expressing plasmid into HeLa cells. While eukaryotic translation initiation factor 4G (eIF4G), which is a known substrate of 2A^pro^, was cleaved in the 2A^pro^-expressing cells, we did not observe PMLIII or PMLIV degradation in the 2A^pro^-expressing cells (Figure 8A, left panel). By contrast, the 3C^pro^ expression resulted in a dramatic reduction in the isoforms PMLIII and IV (Figure 8A, right panel). To investigate whether the 3C^pro^-mediated PML cleavage is specific, we evaluated the impact of the 3C^pro^ inhibitor rupintrivir. As shown in Figure 8B, the GFP-3C-mediated PMLIII and IV degradation in the HeLa cells (Lanes 2 and 3) was blocked by rupintrivir (Lanes 4 and 5 compared with Lanes 2 and 3, respectively). As expected, epoxomicin had no effect and did not inhibit the 3C^pro^-mediated PML degradation (Figure S4 in Supplementary Material, Lanes 5 and 6 compared with Lanes 2 and 3, respectively). Thus, the PML degradation is independent of the proteasome pathway and dependent on 3C protease activity.

After 293T cells overexpressing the isoform PMLIII or PMLIV were transfected with a plasmid encoding GFP-tagged 3C^pro^, the isoforms PMLIII and/or PMLIV were degraded in the 3C-expressing cells but not in the cells transfected with the control plasmid (Figure 8C, Lane 3 or 4 compared with Lane 2). This observation is consistent with a previous report showing that the PML protein was degraded in a 3C^pro^-dependent manner in mengovirus- or EMCV-infected cells (41). In addition, in the Western blot analysis, 3C^pro^ drastically reduced the endogenous PMLIII and PMLIV expression in the IFN-treated cells, while the control pEGFP-C1 had a minimal effect on the IFN-treated cells (Figure 8D). Moreover, the degradation of the other isoforms, i.e., PMLI, II, V, and VI, was also observed as a result of the 3C^pro^ expression (Figure S5 in Supplementary Material). Altogether, 3C^pro^, but not 2A^pro^, induces PML degradation upon EV71 infection.

## Discussion

Previous studies have shown the multi-faceted roles of PML in stress response (46), gene regulation (47), oncogenesis (48), cell senescence (49), DNA damage repair (50), apoptosis (51), and antibacterial (52) and antiviral defense (53). In mammals, a large number of cellular effectors participate in the innate immune response against viruses. Restriction factors that belong to the tripartite motif (TRIM) protein superfamily act as the first line of defense against the incoming pathogens. PML, a member of TRIM family, was initially identified as part of a hybrid protein that contains retinoic acid receptor α and is associated with acute PML (54, 55). As stress-responsive structures, the size and composition of PML-NBs are dynamic depending on the cellular environment (53), which are considered critical for cells to adapt to environmental cues and maintain cellular homeostasis.

Promyelocytic leukemia mediated antiviral responses against diverse cytoplasmic RNA viruses *via* various mechanisms (14–16, 23, 27, 40, 53, 56). An early study by El Asmi et al. showed that PML did not affect VSV entry (23). PML suppressed LCMV transcription by interacting with the Z protein (13). PML and p53 cooperatively participate in antiviral defense during poliovirus infection (14). PMLIV was identified to inhibit VSV replication independent of IRF3 and to promote IFN-β synthesis by increasing IRF3 activation (23). In this report, our findings that PML inhibited EV71 replication provide new insight regarding how hosts and viruses interact. Our study is the first time to show that PML restricted EV71 replication by repressing autophagy. PML playing a regulation of autophagy in viral infection has not been previously reported.

Autophagy is induced in host cells by viruses, including HCMV (57), HCV (58), HSV-1(59), CVB3 (60), influenza A virus (61), HIV-I (62), and EV71 (26, 36). In addition, oxidative stress and reactive oxygen species (ROS) accumulation in cellular display an important role in the stimulation of autophagy under nutrient deficiency (63), hypoxia (64), ischemia injury (65), and other cellular stress conditions (66). Interestingly, intracellular ROS might also serve as a signaling molecule that directly or indirectly activates autophagy by regulating the Akt/mTOR pathway (67–69). In a previous study, EV71-mediated ROS generation in mitochondrial location plays an essential role for viral replication (70). Moreover, PML was shown to function as a stress sensor, and the loss of PML resulted in increased ROS due to impairment in mitochondrial complex II activity (71). AMPK, a cellular energy sensor, was activated in the regulation of ROS production in PML^−/−^ muscle and liver (72). A new study by Niwa-Kawakita et al. identified that PML is an ROS sensor; PML^−/−^ cells accumulate ROS, whereas PML expression decreases ROS levels (73). Therefore, the loss of PML likely triggers ROS production, which may directly or indirectly activate autophagy. Missiroli et al. demonstrated that PML mediated autophagy inhibition by repressing autophagosome formation at mitochondria-associated membranes (39). In this study, the high levels of autophagy in the PML^−/−^ cells were independent of the cellular stress conditions (serum deprivation or viral infection, Figures 4A–C and 5E,F), suggesting that PML plays a vital role in autophagy regulation. In earlier reports, EV71-induced mitochondrial ROS generation could promote viral replication (70, 74) by activating autophagy. Therefore, PML may function as a restriction factor against EV71 replication by repressing autophagy mediated by ROS, while as a countermeasure the 3C^pro^-mediated PML degradation may increase ROS production, which could activate autophagy to support viral replication.

Type I IFNs play an important role in the innate immune response and have been considered the first defense against EV71 infection (75, 76). Moreover, PML gene expression increased in response to IFNs (6) since PML promoter contains IFN-α-stimulated response elements and IFN-γ activation sites, suggesting that PML is a downstream effector of IFNs (2). In previous studies, IFN treatment inhibited EV71 infection; however, the precise mechanisms involved in the antiviral response and particularly the IFN effectors implicated in the inhibition effect are not completely understood. To date, two IFN mediators, i.e., MxA and PKR, have been shown to exert antiviral effects against EV71 infection (77, 78). In our study, PML, an IFN mediator, not only inhibited EV71 replication by itself but also mediated the antiviral activity of IFN-β since this activity was abrogated in the PML^−/−^ HeLa cells (Figure 5D), suggesting that PML plays important roles in the IFN-β antiviral activity against EV71 infection.

According to our data, IFN-β upregulates the PML gene, resulting in increased expression of PMLIII and/or IV isoforms (Figure 5A). IFN-β was significantly less effective in inhibiting EV71 replication when cellular PML was downregulated (Figures 5B,C) and contributed to an enhanced antiviral innate response with the overexpression of the PMLIII or PMLIV isoforms in the PML^−/−^ cells (Figure 5E). Our observation is consistent with an earlier report showing that IFN-β is more effective in blocking HSV-1 replication in murine and human PML^+/+^ cells than in PML^−/−^ cells (79, 80). We hypothesize that the IFN-β-mediated antiviral effect was caused by the induction of downstream antiviral effectors, including PML acting as an ISG downstream of IFN. Overexpression of PMLIV but not III in PML^−/−^ cells upregulated IFN-β (data not shown) though both PMLIII and IV inhibited viral replication (Figure 5E) and exogenous IFN further enhanced the antiviral activity mediated by the overexpressed PMLIII or PMLIV in PML^−/−^ cells. These observations may suggest that PMLIII or IV has both intrinsic antiviral activity and is a critical ISG for IFN-β. Alternatively, PML-NBs may act as cellular functional structures for other ISGs to mediate the IFN-β antiviral statues. The IFNs have three effectors, i.e., PML, MxA, and PKR, in the downstream signaling against EV71 infection though we do not know the detailed molecular events involving MxA and PKR and their relationship with PML. MxA and PKR could be recruited to the PML-NB structures and participate in transmitting antiviral signaling. Alternatively, EV71 infection may downregulate MxA and PKR as a result of elF2α phosphorylation, which makes PML especially critical for mediating IFN-β-mediated antiviral effect since PML was not regulated *de novo* during EV71 infection (Chen et al., unpublished data). In addition, EV71 can inhibit the host innate defense by blocking IFN synthesis or suppressing RIG-I-mediated type I IFN responses through 3C^pro^ (81, 82). Because 3C^pro^ inhibits the type 1 IFN response, we cannot exclude the possibility that in addition to the PML molecule, 3C^pro^ may interfere with the expression of other IFN-stimulatory genes. EMCV was shown to induce PML degradation by altering its localization and reducing PML, which was mediated by 3C^pro^ (41). In this study, we showed that PML was degraded by 3C^pro^ without the modulation of PML mRNA transcription (Figure 8). However, PML reduction may also result from the viral inhibition of cellular translational machinery because 2A^pro^ cleaves the eIF4G (83), which leads to host translational shutoff and results in reduced IFN expression and antiviral ISG proteins (such as the PML protein).

Promyelocytic leukemia acts as a first line of host defense in response to viral infections; however, viruses have evolved ways to counteract PML-mediated antiviral activities (53, 84, 85). PML from the PML-NBs is delocalized to the cytoplasm upon LCMV infection and this delocalization is mediated by the virus-encoded small, non-structural protein Z. During LCMV infection, both PML and protein Z interact with the elongation factor eIF4E, reducing its affinity to the 5′-mRNA cap structure and inhibiting cellular translational machinery (86). The C-terminal region of the protein P encoded by rabies virus can directly interact with the RING finger of PML, resulting in the cytoplasmic translocation of PML (84). In addition, PML-NB size is increased in both infected cells and cells that express P3, an N-terminal truncated version of the phosphoprotein P. The P3 is believed to mediate the enlargement of PML-NBs during infection (84). EMCV infection induces the transfer of PML to the nuclear matrix and leads to an increased PML-NB size. However, this process mediates PML degradation in a proteasome pathway or 3C^pro^-dependent manner (41). EV71 3C^pro^ is a virus-coded protein essential for replication (87, 88) that inhibits antiviral immunity (42–44, 82). In a previous study, 3C^pro^ could cleave CstF-64 (42) and suppress cytokine expression by cleaving the TAK1 complex proteins (82). 3C^pro^ may cleavage TRIF by impairing IFN production in response to TLR3 activation (43). Lei et al. reported that EV71 reduced IRF7 expression in infected cells, which requires 3C^pro^ to cleave IRF7 independent of caspase-3, proteasome, lysosome, and autophagy (43, 44). Therefore, the degradation of PML by 3C^pro^ may represent a new mechanism by which the virus escapes cellular antiviral responses.

## Author Summary

In this article, we describe the roles of PML as a cellular restriction factor in EV71 infection. PML inhibited EV71 replication by inhibiting autophagy in the infected cells, and PML deficiency triggered autophagy, which facilitated viral replication. In addition, the IFN-β-mediated inhibition of EV71 replication required the presence of PML, suggesting that the ISGs likely require the presence of PML for the establishment of an antiviral status. Moreover, the EV71 infection induced PML degradation, which was mediated by virus protease 3C^pro^ independent of the proteasome pathway. These findings demonstrate the important roles of PML in innate immune defense against EV71 and describe a novel mechanism by which the virus evades innate immunity.

## Author Contributions

DC, CF, and XT performed the experiments. DC analyzed the data and wrote the manuscript. ZW and NZ monitored and revised the manuscript.

## Conflict of Interest Statement

The authors have declared that no competing interests exist.

## Supplementary Material

Figure S1 EV71 VP1 synthesis in infected cells overexpressing individual PML isoforms. **(A)** Expression of individual PML isoforms in PML^−/−^ HeLa cells transfected with plasmids expressing FLAG-PMLI, FLAG-PMLII, FLAG-PMLIII, FLAG-PMLIV, FLAG-PMLV, or FLAG-PMLVI as revealed by Western blotting using anti-FLAG and anti-GAPDH antibodies. **(B)** PML^−/−^ HeLa cells transfected with plasmids expressing each PML isoform were infected with EV71 at an MOI of 5 for 24 h. The extracts from these cells, i.e., non-infected (−) or infected (+), were analyzed by performing a Western blot analysis using anti-VP1 and anti-GAPDH antibodies.

Figure S2 Immunofluorescence analysis of infected cells expressing individual PML isoforms. HeLa cells transfected with an empty vector (pEGFP-C1) or a plasmid expressing EGFP-PMLI, EGFP-PMLII, EGFP-PMLIII, EGFP-PMLIV, EGFP-PMLV, or EGFP-PMLVI were infected with EV71 at an MOI of 5 for 12 h. A double immunofluorescence analysis was performed using a monoclonal antibody specific for VP1 (red) and GFP-PMLI and GFP-PMLVI (green). The samples were examined under a fluorescence microscope, and the images were analyzed under an Olympus FluoView FV10i confocal microscope (Tokyo, Japan).

Figure S3 Western blot analysis of uninfected or infected cells expressing VP1 and LC3-I and II in siATG5-treated or siNC-treated cells. HeLa WT cells were transfected with si-ATG5 or negative siRNA (siNC) and then with GLuc-EV71 at an MOI of 5 for 48 h. The cells were harvested, and the cell lysates were analyzed by performing a Western blot analysis using antibodies against ATG5, LC-3B, VP1, and GAPDH. The density of the bands was scanned by densitometry and expressed relative to that of the siNC-treated and mock-infected cells. The siNC-treated and mock-infected cells were assigned a value of 1.00. ****p* < 0.005, ***p* < 0.01.

Figure S4 Effect of the proteasome inhibitor epoxomicin on the degradation of PMLIII and IV. HeLa cells were transfected with the control plasmid EGFP-C1 or increasing amounts of GFP-3C. At 24 h post transfection, the cells were treated with epoxomicin (1 μM) for 24 h. Then, the cell lysates were processed for a Western blot analysis. The density of the bands was scanned by performing densitometry and expressed relative to the control (pEGFP-C1), which was assigned a value of 1.00. ****p* < 0.005.

Figure S5 PML^−/−^ HeLa cells were transfected with plasmids expressing individual FLAG-PML isoforms for 24 h. Cells expressing each individual PML isoform were transfected with an empty control pEGFP-C1 or pEGFP-3C for 24 h. The cell lysates were analyzed by performing a Western blot analysis using antibodies specific for FLAG, GFP, and GAPDH. Each experiment was performed three times, and representative results are shown.
