# Supplementary figures and images for: Promyelocytic Leukemia Restricts Enterovirus 71 Replication by Inhibiting Autophagy
Source: Front Immunol. 2018 Jun 5;9:1268. doi: 10.3389/fimmu.2018.01268 (PMC5996053; doi:10.3389/fimmu.2018.01268)

Figure S1

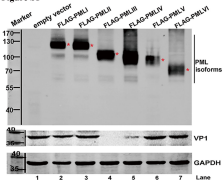

Supplement: Figure S1 — EV71 VP1 synthesis in infected cells overexpressing individual PML isoforms. (A) Expression of individual PML isoforms in PML−/− HeLa cells transfected with plasmids expressing FLAG-PMLI, FLAG-PMLII, FLAG-PMLIII, FLAG-PMLIV, FLAG-PMLV, or FLAG-PMLVI as revealed by Western blotting using anti-FLAG and anti-GAPDH antibodies. (B) PML−/− HeLa cells transfected with plasmids expressing each PML isoform were infected with EV71 at an MOI of 5 for 24 h. The extracts from these cells, i.e., non-infected (−) or infected (+), were analyzed by performing a Western blot analysis using anti-VP1 and anti-GAPDH antibodies. [file image_1.PDF]

Figure S2

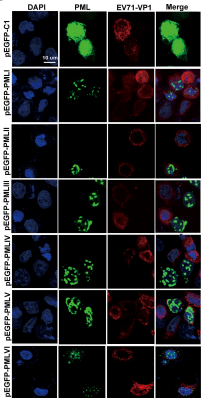

Supplement: Figure S2 — Immunofluorescence analysis of infected cells expressing individual PML isoforms. HeLa cells transfected with an empty vector (pEGFP-C1) or a plasmid expressing EGFP-PMLI, EGFP-PMLII, EGFP-PMLIII, EGFP-PMLIV, EGFP-PMLV, or EGFP-PMLVI were infected with EV71 at an MOI of 5 for 12 h. A double immunofluorescence analysis was performed using a monoclonal antibody specific for VP1 (red) and GFP-PMLI and GFP-PMLVI (green). The samples were examined under a fluorescence microscope, and the images were analyzed under an Olympus FluoView FV10i confocal microscope (Tokyo, Japan). [file image_2.PDF]

Figure S3

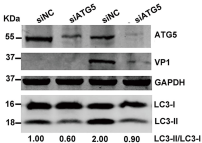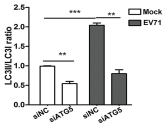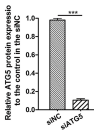

Supplement: Figure S3 — Western blot analysis of uninfected or infected cells expressing VP1 and LC3-I and II in siATG5-treated or siNC-treated cells. HeLa WT cells were transfected with si-ATG5 or negative siRNA (siNC) and then with GLuc-EV71 at an MOI of 5 for 48 h. The cells were harvested, and the cell lysates were analyzed by performing a Western blot analysis using antibodies against ATG5, LC-3B, VP1, and GAPDH. The density of the bands was scanned by densitometry and expressed relative to that of the siNC-treated and mock-infected cells. The siNC-treated and mock-infected cells were assigned a value of 1.00. ***p < 0.005, **p < 0.01. [file image_3.PDF]

**Figure S4**

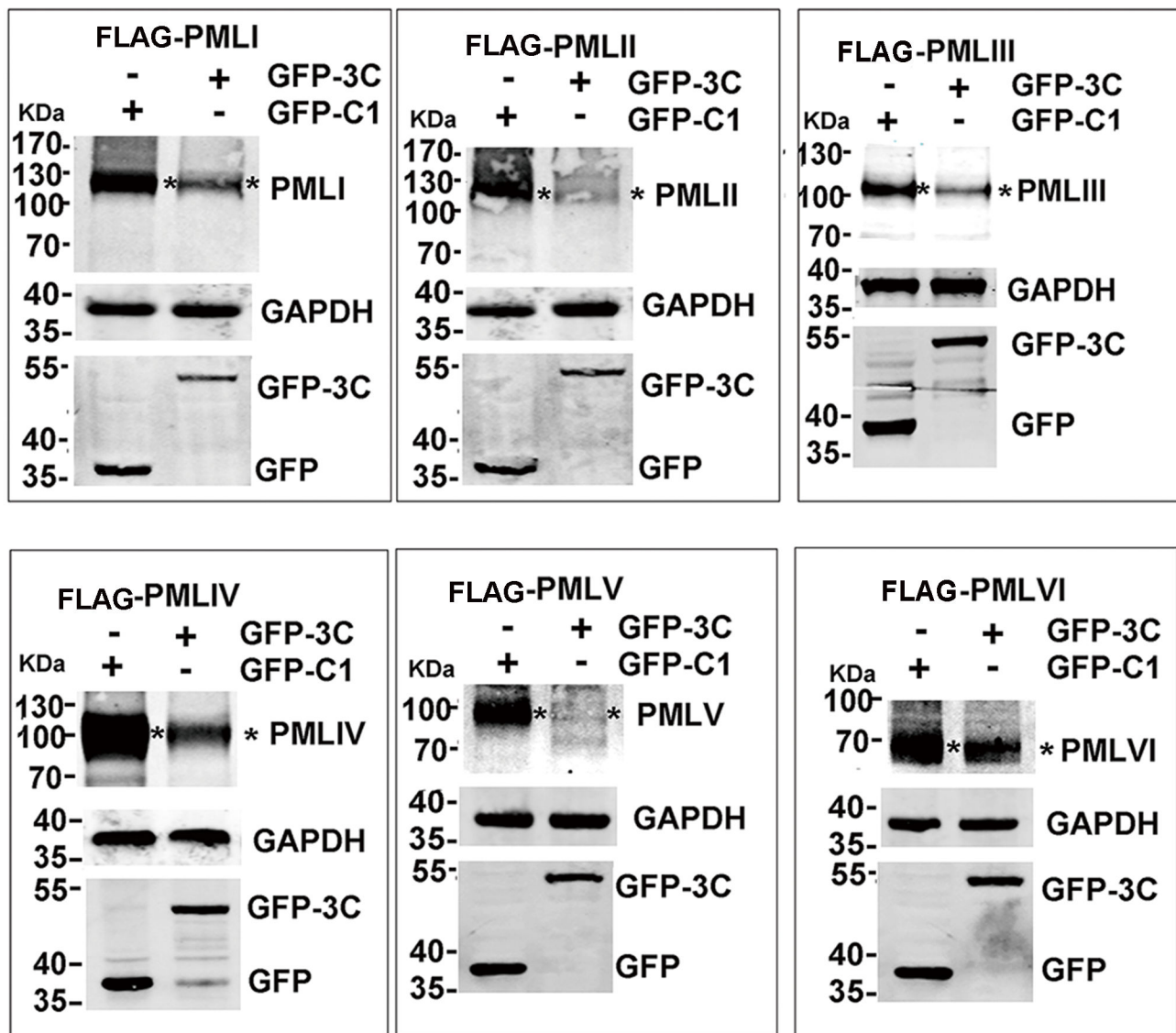

Supplement: Figure S4 — Effect of the proteasome inhibitor epoxomicin on the degradation of PMLIII and IV. HeLa cells were transfected with the control plasmid EGFP-C1 or increasing amounts of GFP-3C. At 24 h post transfection, the cells were treated with epoxomicin (1 μM) for 24 h. Then, the cell lysates were processed for a Western blot analysis. The density of the bands was scanned by performing densitometry and expressed relative to the control (pEGFP-C1), which was assigned a value of 1.00. ***p < 0.005. [file image_4.PDF]

**Figure S5**

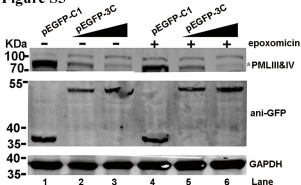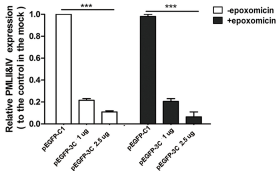

Supplement: Figure S5 — PML−/− HeLa cells were transfected with plasmids expressing individual FLAG-PML isoforms for 24 h. Cells expressing each individual PML isoform were transfected with an empty control pEGFP-C1 or pEGFP-3C for 24 h. The cell lysates were analyzed by performing a Western blot analysis using antibodies specific for FLAG, GFP, and GAPDH. Each experiment was performed three times, and representative results are shown. [file image_5.PDF]
